# Prevalence, Risk Factors, and Genetic Characterization of Extended-Spectrum Beta-Lactamase *Escherichia coli* Isolated From Healthy Pregnant Women in Madagascar

**DOI:** 10.3389/fmicb.2021.786146

**Published:** 2021-12-24

**Authors:** Milen Milenkov, Saida Rasoanandrasana, Lalaina Vonintsoa Rahajamanana, Rivo Solo Rakotomalala, Catherine Ainamalala Razafindrakoto, Christian Rafalimanana, Emile Ravelomandranto, Zakasoa Ravaoarisaina, Emilie Westeel, Marie Petitjean, Jimmy Mullaert, Olivier Clermont, Laurent Raskine, Luc Hervé Samison, Hubert Endtz, Antoine Andremont, Erick Denamur, Florence Komurian-Pradel, Laurence Armand-Lefevre

**Affiliations:** ^1^Fondation Mérieux, Lyon, France; ^2^Université de Paris, IAME, INSERM UMR 1137, Paris, France; ^3^Laboratoire de Bactériologie, CHU Joseph Raseta Befelatanana, RESAMAD Network, Antananarivo, Madagascar; ^4^Laboratoire de Bactériologie, CHU Mère-Enfant Tsaralalana, RESAMAD Network, Antananarivo, Madagascar; ^5^Laboratoire de Bactériologie, CHU PZaGa Androva, RESAMAD Network, Mahajanga, Madagascar; ^6^Laboratoire de Bactériologie, CHU Morafeno, RESAMAD Network, Toamasina, Madagascar; ^7^Laboratoire de Bactériologie, CHU Joseph Ravoahangy Andrianavalona, RESAMAD Network, Antananarivo, Madagascar; ^8^Laboratoire de Bactériologie, CHRR Alaotra Mangoro, RESAMAD Network, Ambatondrazaka, Madagascar; ^9^CHU Mère Enfant Ambohimiandra, RESAMAD Network, Antananarivo, Madagascar; ^10^Centre d’Infectiologie Charles Mérieux, University of Antananarivo, Antananarivo, Madagascar; ^11^Department of Medical Microbiology and Infectious Diseases, Erasmus MC, Rotterdam, Netherlands; ^12^Laboratoire de Génétique Moléculaire, Hôpital Bichat-Claude Bernard, AP-HP Nord-Université de Paris, Paris, France; ^13^Laboratoire de Bactériologie, Hôpital Bichat-Claude Bernard, AP-HP Nord-Université de Paris, Paris, France

**Keywords:** extended-spectrum beta-lactamase (ESBL), *E. coli*, digestive carriage, epidemiology, whole genome sequencing, Madagascar

## Abstract

Antimicrobial resistance is a major public health concern worldwide affecting humans, animals and the environment. However, data is lacking especially in developing countries. Thus, the World Health Organization developed a One-Health surveillance project called Tricycle focusing on the prevalence of ESBL-producing *Escherichia* co*li* in humans, animals, and the environment. Here we present the first results of the human community component of Tricycle in Madagascar. From July 2018 to April 2019, rectal swabs from 492 pregnant women from Antananarivo, Mahajanga, Ambatondrazaka, and Toamasina were tested for ESBL-*E. coli* carriage. Demographic, sociological and environmental risk factors were investigated, and *E. coli* isolates were characterized (antibiotic susceptibility, resistance and virulence genes, plasmids, and genomic diversity). ESBL-*E. coli* prevalence carriage in pregnant women was 34% varying from 12% (Toamasina) to 65% (Ambatondrazaka). The main risk factor associated with ESBL*-E. coli* carriage was the rainy season (OR = 2.9, 95% CI 1.3–5.6, *p* = 0.009). Whole genome sequencing was performed on 168 isolates from 144 participants. *bla*_CTX–M–15_ was the most frequent ESBL gene (86%). One isolate was resistant to carbapenems and carried the *bla*_*NDM–*5_ gene. Most isolates belonged to commensalism associated phylogenetic groups A, B1, and C (90%) and marginally to extra-intestinal virulence associated phylogenetic groups B2, D and F (10%). Multi locus sequence typing showed 67 different sequence types gathered in 17 clonal complexes (STc), the most frequent being STc10/phylogroup A (35%), followed distantly by the emerging STc155/phylogroup B1 (7%), STc38/phylogroup D (4%) and STc131/phylogroup B2 (3%). While a wide diversity of clones has been observed, SNP analysis revealed several genetically close isolates (*n* = 34/168) which suggests human-to-human transmissions. IncY plasmids were found with an unusual prevalence (23%), all carrying a *bla*_CTX–M–15_. Most of them (85%) showed substantial homology (≥85%) suggesting a dissemination of IncY ESBL plasmids in Madagascar. This large-scale study reveals a high prevalence of ESBL-*E. coli* among pregnant women in four cities in Madagascar associated with warmth and rainfall. It shows the great diversity of *E. coli* disseminating throughout the country but also transmission of specific clones and spread of plasmids. This highlights the urgent need of public-health interventions to control antibiotic resistance in the country.

## Introduction

Antimicrobial resistance (AMR) has become a tremendous global public health issue, which affects humans, animals and the environment. One of the major threats today is the worldwide dissemination of multi-drug resistant bacteria, particularly the extended-spectrum beta-lactamase (ESBL)-producing *Enterobacterales* (ESBL-E) (Lynch et al., 2013). Indeed, since the 2000s the incidence of ESBL-E has been steadily increasing in the community especially in middle- and low-income countries (Woerther et al., 2013). A meta-analysis conducted in 2016 estimated that 14% of healthy individuals worldwide were carriers of ESBL-E (Karanika et al., 2016). However, large regional disparities can be observed, with an estimated prevalence of 5% in Europe, 20% around the Mediterranean basin and in Africa, 50% in the Pacific and 70% in South-East Asia (Woerther et al., 2011; Karanika et al., 2016). An important cornerstone in the control of AMR is the implementation and monitoring of AMR surveillance indicators [World Health Organization [WHO], 2015]. In this context, the World Health Organization (WHO) proposed a surveillance protocol called Tricycle aiming to provide a simplified, integrated and *trans*-sectoral surveillance system. The proposed surveillance focuses on a single key indicator, the prevalence of ESBL-*Escherichia coli*, in the three major settings that are the human (community carriage and hospital infection), the food-chain (poultry) and the environment (water). *E. coli* was chosen as the target organism because it is a commensal of the gut of all humans and a wide variety of warm-blooded animals, and is also widespread in the environment (Savageau, 1983), but primarily because infections with ESBL-*E. coli* in humans, result in severe morbidity and mortality (Schwaber and Carmeli, 2007). For the human component of the Tricycle protocol, the population targeted as representative of the community was healthy pregnant women approaching or at delivery.

Thanks to a structured laboratory network (RESAMAD), the Tricycle protocol was implemented in Madagascar in 2018 by Fondation Mérieux to support the implementation of a functional National Action Plan on AMR. The need for such a surveillance system in the country was urgent as data on AMR were sparce and the few studies conducted previously reported increasing ESBL-E rates in the community, going from 10.1% in 2010 (Herindrainy et al., 2011) to 18.5% in 2014 (Chereau et al., 2015).

Here we report the first results from the human community component of the Tricycle protocol in Madagascar, describing ESBL-*E. coli* carriage and its associated risk factors among healthy pregnant women. We also investigated the genomic characteristics of isolated strains such as presence of resistance and virulence genes, plasmids and their genomic diversity.

## Materials and Methods

### Study Design

**FIGURE 1:**
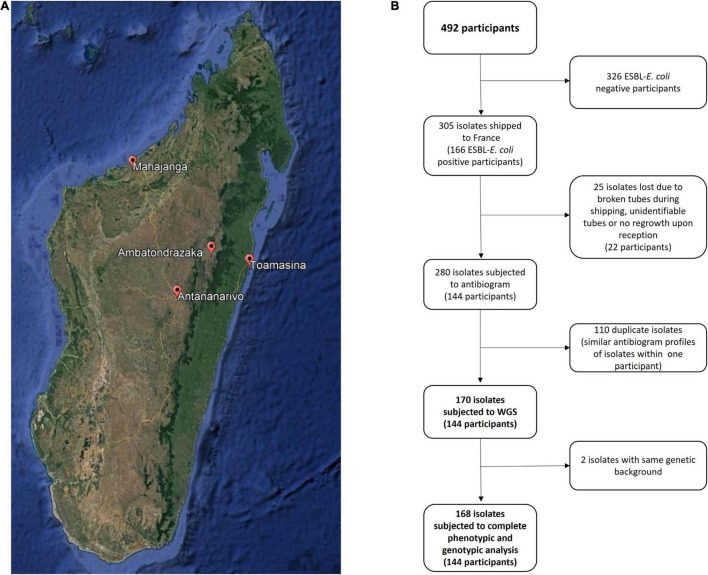
Study characteristics. **(A)** Geographical situation of cities involved in the project in Madagascar. **(B)** Flow chart representing the evolution of the number of isolates along study progress.

The present study was conducted in the context of the Tricycle *trans*-sectoral surveillance project, approved by the Ethics Committee of Ministry of Health, Madagascar (038-MSANP/CERBM). Healthy pregnant women were enrolled from July 2018 to April 2019 after signing a written informed consent. Rectal swabs were collected at the last prenatal consultation in three maternity wards in the capital city Antananarivo [Joseph Raseta Befelatanana Hospital (JRB) – July–August 2018, Mère-Enfant Tsaralalana Hospital (TSA) – July–October 2018, and Joseph Ravoahangy Andrianavalona Hospital (JRA) – March–April 2019] as well as three in provincial cities [Mahajanga Androva Hospital (MAH) – September–November 2018], Toamasina [Morafeno Hospital (TOA) – August–November 2018] and Ambatondrazaka [Alaotra Mangoro Hospital (AMB) December 2018 to March 2019] (Figure 1A). The laboratories of all these 6 hospitals have been included in the study as they are part of the RESAMAD network, Madagascar and have a history of working with shared protocols.

### Microbiology and Resistance Profile Assessment

In each hospital laboratory, samples were plated immediately on MacConkey Agar supplemented with 4 g/L cefotaxime and cultured overnight at 35°C ± 2°C. For each plate showing growth, three separate *E. coli* presumptive colonies were selected and tested for indole production. Confirmation of ESBL production was performed by double disk synergy testing (amoxicillin/clavulanic acid, cefotaxime, cefepime, and ceftazidime). Indole positive isolates showing a synergy between amoxicillin/clavulanic acid and cephalosporins were stored in stock culture agar (Bio-Rad, Marnes-La-Coquette, France) and shipped to France (World Health Organization [WHO], 2021).

At Fondation Mérieux (Lyon, France) the identification of the indole-positive isolates was verified on a Vitek 2 Compact system (bioMérieux, Marcy-l’Etoile, France). Antimicrobial susceptibility was determined by the disk diffusion method on Mueller-Hinton agar according to CASFM/EUCAST recommendations (European Committee on Antimicrobial Susceptibility Testing, Clinical breakpoints, 2019). The following antimicrobial agents were tested: amoxicillin, amoxicillin/clavulanic acid, ticarcillin, ticarcillin/clavulanic acid, piperacillin, piperacillin/tazobactam, cefotaxime, cefalexin, ceftazidime, cefepime, cefoxitin, aztreonam, temocillin, mecillinam, imipenem, meropenem, ertapenem, fosfomycin, colistin, chloramphenicol, nalidixic acid, ofloxacin, ciprofloxacin, levofloxacin, amikacin, gentamicin, netilmicin, tobramycin, tetracycline, sulfonamides, trimethoprim, and trimethoprim/sulfamethoxazole.

### Epidemiological Data Collection

The following data were collected: age, address (limited to the district to preserve anonymity), level of education, access to electricity, water and toilets, presence of pet or farm animals, recent intake of antibiotics (in the last 3 months). Rainfall and mean temperatures corresponding to the time of sampling in each sampling location, were retrieved from https://www.historique-meteo.net/afrique/madagascar.

### Whole Genome Sequencing

#### Illumina Technology

For each pregnant woman, all isolates with distinct antibiotic profiles, based on the susceptibility of 32 antibiotics on the antibiogram, were selected for whole genome sequencing (WGS). Nucleic acid extraction was performed using the QIAamp DNA Mini Kit (Qiagen, Les Ulis, France). Whole genome sequencing was performed on a NextSeq platform (Illumina, San Diego, CA, United States) using the Celero DNA-Seq PCR free kit (NuGen Technologies, Redwood, CA, United States) for library preparation and on a MiniSeq system (Illumina) using the NextEra DNA Flex Library Prep kit for library preparation (Illumina). Contigs were assembled with SPAdes genome assembler v.3.11.0 and contigs longer than 500 bp were further analyzed. Serotypes and Sequence types (ST) were determined using SerotypeFinder v.1.0.0 (Joensen et al., 2015) and MLST Finder v1.8 (Larsen et al., 2012) (Warwick University ST scheme), respectively (from the Center for Genomic Epidemiology)^1^. ST complexes (STc) were determined using the EnteroBase^2^. Identification of resistance genes and plasmid replicons was performed using ResFinder v.2020-10-20 (Zankari et al., 2012) (with 90% threshold ID and 60% minimum overlapping gene length) and PlasmidFinder v.2020-07-13 (Carattoli et al., 2014) (with 95% threshold ID and 60% minimum overlapping gene length), respectively. Chromosomal or plasmid localization of ESBL encoding genes was predicted using PlaScope v1.3.1 which compares contigs from an assembly to complete chromosome and plasmid sequences from curated databases and then classifies them into three categories: “chromosome,” “plasmid,” and “unclassified” (Royer et al., 2018). Phylogroups were determined using the standalone version of the ClermonTyping program (Beghain et al., 2018). Virulence genes were detected using the Abricate software (v.0.9.8) (Bourrel et al., 2019) to query Virulence Finder v. 2016-02-18 (Joensen et al., 2014) and VfDB v. 2018-11-05 (Liu et al., 2019) databases and an in house developed virulence factors database (Bourrel et al., 2019). A minimum spanning tree was constructed using the MSTreeV2 algorithm of GrapeTree software v. 1.5.0 (Zhou et al., 2018). Roary software v3.12 (Page et al., 2015) and PhyML v3.3.20180621 (Guindon et al., 2010) (GTR model, estimated proportion of invariable sites and 1000 bootstraps) were used to construct a maximum likelihood phylogenetic tree. A maximum-likelihood phylogenetic tree was built using the complete genome of *Escherichia fergusonii* strain ATCC35471 (GenBank accession number: GCA_012811895.1) as the root. A maximum-likelihood phylogenetic tree of phylogenetic group A isolates was constructed using Fast hierarchical Bayesian analysis of population structure (fastBAPS) and rooting on the *E. coli* B1 strain 55989 (GenBank accession number: GCA_000026245.1). Figures were generated using iTOL v6.3 (Letunic and Bork, 2007). SNPs were calculated by Parsnp v1.12 (Treangen et al., 2014). Following the literature, a cut-off of 10 SNPs was chosen to consider isolates as closely related (Schürch et al., 2018).

#### Oxford Nanopore Technology

Isolates containing IncY replicons were sequenced using Nanopore technology, in order to reconstruct the plasmids by combining the long reads generated by Nanopore and the high accuracy of Illumina reads. Nucleic acid extraction was performed using the MasterPure Complete DNA and RNA Purification kit (Lucigen, Middleton, WI, United States). Libraries were prepared using SQK-LSK109 kit (Oxford Nanopore Technologies, Oxford, United Kingdom). Whole genome sequencing was performed on a MinION platform (Oxford Nanopore Technologies). Plasmid sequences were reconstructed using Illumina and Nanopore reads with Unicycler v0.4.9b software (Wick et al., 2017). All plasmid sequences were compared using BLAST v2.8.0. Annotation was performed using PROKKA v1.16.6 (Seemann, 2014). Graphic presentation was generated with BRIG v0.95 (Alikhan et al., 2011).

### Statistical Analysis

Descriptive statistics included counts and percentages for the description of binary variables, and median and interquartile range for quantitative variables. Factors associated with ESBL-*E. coli* carriage were identified with univariable logistic regressions with carriage as dependent variable. We reported odds-ratio estimates with 95% profile confidence intervals and *p*-values from the likelihood ratio test. In order to take into account center heterogeneity, we also used mixed logistic regression with a random intercept corresponding to the recruiting center (*n* = 6 centers) and estimated with Laplace approximation of likelihood. We also provided profile 95% confidence intervals and *p*-values from the likelihood ratio test. The threshold for statistical significance was 0.05 and all analysis were performed with R software v4.0.5.

To assess the correlation between the phenotypical resistance and the resistant gene content and to visualize co-resistance patterns, a correlation matrix for binary variables was built using the corr.test function (Pearson method) from the psych R package version 2.1.9 (susceptible = 0, intermediate/resistant = 1 for phenotypic resistance and absence = 0, presence = 1 for resistance genes). Correlations were visualized using the corrplot R package version 0.9.

## Results

### Prevalence and Variables Associated With Extended-Spectrum Beta-Lactamase-*E. coli* Carriage

In total, 492 pregnant women were enrolled in the study (Table 1), 289 participants being from the capital and 203 from the study’s provincial cities. Of the total group, 166 (34%) were colonized with ESBL-*E. coli* with significant differences in prevalence between various centers (Table 2).

**TABLE 1:** Characteristics of pregnant women enrolled and of weather conditions during sampling in Madagascar, July 2018 to April 2019 (*n* = 492).

Variable		*N* (%) or med (Q1–Q3)
Age (years, missing = 1)	≤20	71 (14)
	21–25	155 (32)
	26–30	147 (30)
	>30	118 (24)
Site	TOA	66 (13)
	TSA	99 (20)
	JRB	100 (20)
	JRA	90 (18)
	MAH	100 (20)
	AMB	37 (8)
Season	Dry	338 (69)
	Wet	154 (31)
Rainfall (mm)		14 (1–150)
Mean T (°C)		21 (18–27)
Study area	Capital	289 (59)
	Province	203 (41)
Education	Absent or elementary school	63 (13)
	High school graduate	257 (52)
	University graduate	172 (35)
Toilets	Shared	281 (57)
	Private	211 (43)
Electricity access		442 (90)
Drinking water access	Private	140 (29)
(Missing = 1)	Shared	351 (71)
Pets or farm animals		234 (48)
Recent antibiotic intake		70 (14)
ESBL-*E. coli* carriage		166 (34)

*JRB, Antananarivo – Joseph Raseta Befelatanana Hospital; TSA, Antananarivo – Mère-Enfant Tsaralalana Hospital; JRA, Antananarivo – Joseph Ravoahangy Andrianavalona Hospital; MAH, Mahajanga – Androva Hospital; TOA, Toamasina – Morafeno Hospital; and AMB, Ambatondrazaka – Alaotra Mangoro Hospital.*

**TABLE 2:** Factors associated with ESBL-*E. coli* carriage in pregnant women in Madagascar, 2018–2019.

Variable		N carriers/N total (%)	Univariable logistic regression		Mixed logistic regression	
			OR (95% CI)	*p*	OR (95% CI)	*p*
Age (years)	≤20	29/71 (41)	1 (ref)	0.49	1 (ref)	**0.039**
	21–25	54/155 (35)	0.8 (0.4–1.4)		0.8 (0.4–1.5)	
	26–30	45/147 (31)	0.6 (0.4–1.2)		0.5 (0.2–0.9)	
	>30	38/118 (32)	0.7 (0.4–1.3)		0.5 (0.2–0.9)	
Site	TOA	8/66 (12)	1 (ref)	**<0.001**		
	TSA	21/99 (21)	2.0 (0.8–5.0)			
	JRB	24/100 (24)	2.3 (1.0–5.8)			
	JRA	41/90 (46)	6.1 (2.7–15.1)			
	MAH	48/100 (48)	6.7 (3.0–16.5)			
	AMB	24/37 (65)	13.4 (5.1–38.5)			
Study area	Capital	86/289 (30)	1 (ref)	**0.026**		
	Province	80/203 (39)	1.5 (1.1–2.2)			
Season	Dry	85/338 (25)	1 (ref)	**<0.001**	1 (ref)	**0.009**
	Wet	81/154 (53)	3.3 (2.2–4.9)		2.9 (1.3–5.6)	
Rainfall (per 100 mm)			1.4 (1.2–1.5)	**<0.001**	1.2 (1.0–1.3)	0.056
mean T (°C)			1.1 (1.1–1.2)	**<0.001**	1.2 (1.0–1.4)	**0.018**
Education	Absent or elementary	15/63 (24)	1 (ref)	0.077	1 (ref)	0.68
	Secondary	84/257 (33)	1.6 (0.8–3.0)		1.2 (0.6–2.5)	
	University or graduate	67/172 (39)	2.0 (1.1–4.0)		1.4 (0.7–2.9)	
Toilets	Shared	90/281 (32)	1 (ref)	0.35	1 (ref)	0.54
	Private	76/211 (36)	1.2 (0.8–1.7)		0.9 (0.6–1.3)	
Electricity access	No	22/48 (46)	1 (ref)	0.066	1 (ref)	**0.046**
	Yes	143/442 (32)	0.6 (0.3–1.0)		0.5 (0.3–1.0)	
Drinking water access	Private	51/140 (36)	1 (ref)	0.44	1 (ref)	0.49
	Shared	115/351 (33)	0.9 (0.6–1.3)		1.2 (0.8–1.8)	
Pets or farm animals	No	84/258 (33)	1 (ref)	0.56	1 (ref)	0.75
	Yes	82/234 (35)	1.1 (0.8–1.6)		1.1 (0.7–1.6)	
Recent antibiotic intake	No	140/421 (33)	1 (ref)	0.69	1 (ref)	0.87
	Yes	25/70 (36)	1.1 (0.6–1.9)		1.0 (0.6–1.8)	

*JRB, Antananarivo – Joseph Raseta Befelatanana Hospital; TSA, Antananarivo – Mère-Enfant Tsaralalana Hospital; JRA, Antananarivo – Joseph Ravoahangy Andrianavalona Hospital; MAH, Mahajanga – Androva Hospital; TOA, Toamasina – Morafeno Hospital; and AMB: Ambatondrazaka – Alaotra Mangoro Hospital. Significant p values are in bold.*

#### Variables Associated With Extended-Spectrum Beta-Lactamase-*E. coli* Carriage

In Antananarivo, prevalence in the three maternity wards differed, ranging from 21% (TSA Hospital) to 46% (JRA Hospital). Comparing ESBL-*E. coli* prevalence by city, prevalence was highest in Ambatondrazaka (65%, 24/37), followed by Mahajanga (48%, 48/100), Antananarivo (29.8%, 86/289) and Toamasina (12%, 8/66), (*p* < 0.001). Other factors associated with ESBL-*E. coli* carriage from the univariable analysis were sampling season (OR = 3.3 for the wet season, 95% CI 2.2–4.9, *p* < 0.001), rainfall (OR = 1.4, 95% CI 1.2–1.5, *p* < 0.001) and temperature (OR = 1.1 95% CI 1.1–1.2, *p* < 0.001). After taking into account center heterogeneity with mixed logistic regression, the association of ESBL-*E. coli* carriage with the wet season (OR = 2.9, 95% CI 1.3–5.6, *p* = 0.009), warmth (OR = 1.2 per°C, 95% CI 1.0–1.4, *p* = 0.018) and rainfall (OR = 1.2 per 100 mm, 95% CI 1.0–1.3, *p* = 0.056 borderline significant), was confirmed (Table 2). Sensitivity analysis with mixed logistic regression also revealed that older age and access to electricity were associated with a lower risk of carriage (*p* = 0.039 and 0.046, respectively). Other associations were only slightly modified (Table 2).

### Extended-Spectrum Beta-Lactamase-*E. coli* Characterization

Among the 305 isolates received in France from the 166 ESBL-*E. coli* positive pregnant women, 280 were subjected to antibiotic susceptibility testing, of which, 112 were considered duplicates. In all, 168 isolates from 144 participants were selected for further phenotypic and genomic analysis (Figure 1B).

#### Antibiotic Resistance

**FIGURE 2:**
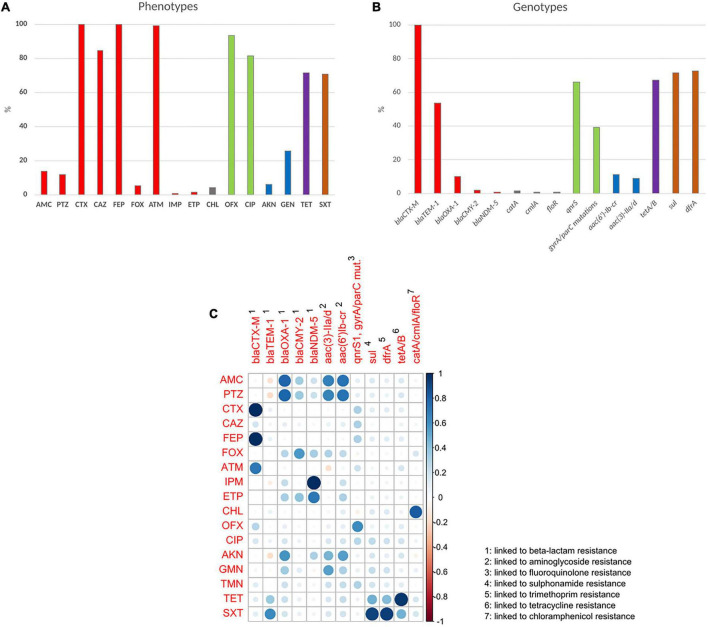
Prevalence of phenotypic antibiotic resistance and correlation to resistance genotype. **(A)** Prevalence of phenotypic antibiotic resistance. **(B)** Prevalence of antibiotic resistance genes. Percentages correspond to resistant and intermediately resistant isolates for each antibiotic. Red, beta-lactams; blue, aminoglycosides; green, fluoroquinolones; brown, folate synthesis inhibitors. **(C)** Correlation matrix between resistance phenotype and genotype. Blue circles represent significant positive correlations. Red circles represent significant negative correlations. Blank squares represent correlations without statistical significance. Circle size and color darkness represent the value of the correlation coefficient. AMC, amoxicillin/clavulanic acid; PTZ, piperacillin/tazobactam; CTX, cefotaxime; CAZ, ceftazidime; FEP, cefepime; FOX, cefoxitin; ATM, aztreonam; IMI, imipenem; ETP, ertapenem; CHL, chloramphenicol; OFX, ofloxacin; CIP, ciprofloxacin; AKN, amikacin; GEN, gentamicin; TET, tetracycline; SXT, sulfamethoxazole/Trimethoprim.

Concerning beta-lactams, 11.9% (*n* = 20) of the isolates were resistant or showed intermediate susceptibility to piperacillin/tazobactam, and 0.6% (*n* = 1) to imipenem. Concerning other antibiotic classes, 6% (*n* = 10) of the isolates were resistant or intermediately resistant to amikacin, 25.6% (*n* = 43) to gentamicin, 70.8% (*n* = 119) to co-trimoxazole and 82% (*n* = 137) to ciprofloxacin (Figure 2A and Supplementary Table 1).

#### Resistance Genes

The ESBL phenotype was due in all cases to a *bla*_CTX–M_ gene (Figure 2B) with 89.9% (*n* = 151) belonging to the CTX-M-1 group, 9.5% (*n* = 16) to the CTX-M-9 group, and 0.6% (*n* = 1) to the CTX-M-2 group. CTX-M genes were predicted as chromosomally located in 31% of the isolates (*n* = 52). No TEM or SHV ESBL genes were detected. The most frequent *bla*_*CTX–M*_ gene was by far *bla*_CTX–M–15_ (85.7%, *n* = 144), followed by *bla*_*CTX–M–*27_ (8.3%, *n* = 14), *bla*_*CTX–M–*55_ (2.4%, *n* = 4), *bla*_*CTX–M–*3_ and *bla*_*CTX–M–*14_ (1.2%, *n* = 2 each) and *bla*_*CTX–M–*1_ and *bla*_*CTX–M–*124_ (0.6%, *n* = 1 each). A high proportion of the isolates (54.2%, *n* = 91) carried both a *bla*_*CTX–M*_ gene and the penicillinase *bla*_*TEM–*1*B*_ gene. Three isolates harbored the plasmid borne cephalosporinase gene *bla_*CMY–*2_*. The isolate, resistant to carbapenems, carried the metalo beta-lactamase gene *bla_*NDM–*5_* and was retrieved from a pregnant woman from the city of Mahajanga.

We observed co-resistance to other antibiotic classes in 98% (*n* = 165) of the strains. The most frequent resistance genes associated with the ESBL profile were from *sul* and *dfr* families, identified in 71.4% (*n* = 120) and 72.6% (*n* = 122) of the isolates, respectively (Figure 2B). In 95% of those (*n* = 118) presence of a *sul* gene was associated with that of a *dfr* gene, conferring resistance to trimethoprim/sulfamethoxazole.

The fluoroquinolone resistance gene qnrS1 was identified in a high proportion of the isolates (66.1%, *n* = 111). Other fluoroquinolone resistance mechanisms were point mutations, observed in 39.3% (*n* = 66) of the isolates, mainly gyrA S83L, gyrA D87N and parC S80I (Figure 2B and Supplementary Table 2).

Acquired resistance genes and mutations detected by WGS were strongly correlated with phenotypic resistance (Figure 2C). Some associations were observed because of the co-occurrence of genes on common mobile genetic elements resulting in co-resistance phenotypes.

#### Phylogeny

Most of the isolates (89.9%, *n* = 151) belonged to phylogenetic groups associated with commensalism including A (72%, *n* = 121), B1 (16.1%; *n* = 27) and C (1.8%, *n* = 3) and in much lesser extent to phylogenetic groups associated with extra-intestinal virulence D (6%, *n* = 10), B2 (3%, *n* = 5) and F (1.2%, *n* = 2).

Multi locus sequence typing revealed 63 different sequence types (ST) gathered in 17 clonal complexes (STc), the most frequent being STc10/phylogroup A (35.1% of the isolates, *n* = 59), STc155/phylogroup B1 (6.5%, *n* = 11), STc38/phylogroup D (4.2%, *n* = 7) and STc131/phylogroup B2 (3%, *n* = 5) (Supplementary Table 3). Isolates from STc155 were mainly ST155 (*n* = 8, 73%) and ST58 (*n* = 2, 18%). All isolates from phylogenetic group B2 belonged to ST131, 4/5 were O25:H4 serotype and one O16:H5 serotype.

**FIGURE 3:**
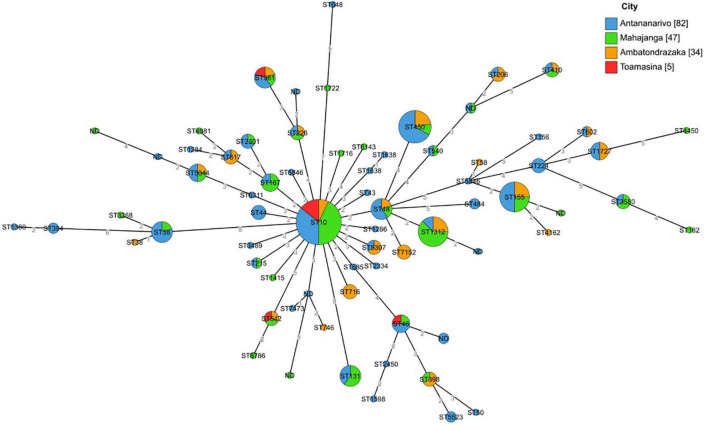
Minimum spanning tree based on MLST analysis of 168 sequenced isolates from pregnant women. Minimum spanning tree colors are coded by the geographical origin of the isolates. Labels indicate the sequence types. Distances are labeled as numbers on the branches.

The ST minimum spanning tree showed important genetic diversity among isolates. No ST clustering was observed related to the geographical origin of the isolates (Figure 3). Mapping of ESBL-*E. coli* carriers residing in Antananarivo (Supplementary Figure 1) did not show geographical ST clustering in the capital either.

**FIGURE 4:**
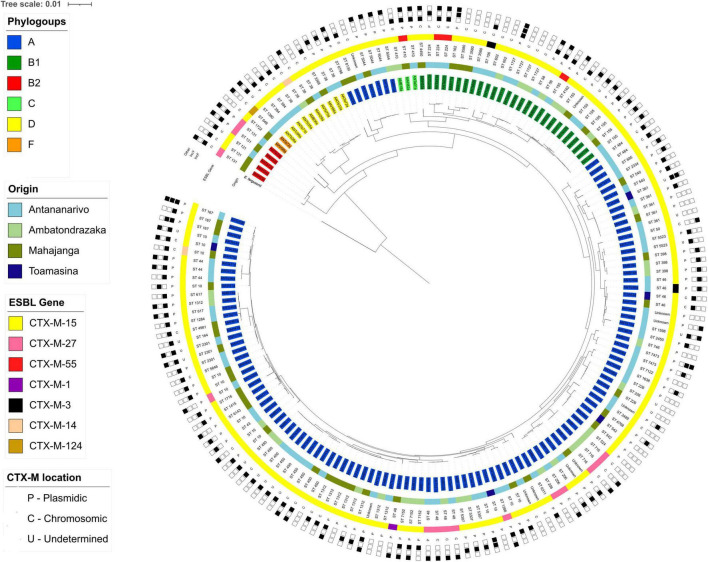
Phylogenetic tree based on core genome sequences of the 168 ESBL*-E. coli* isolates. The tree has been constructed with *E. fergusonii* used as the root. Isolates names are highlighted depending on their phylogenetic group (inner ring). Colored strips represent the geographical origin of the isolates (ring two) and the CTX-M enzyme detected (ring four). STs are given in the third ring. The penultimate ring represents the genomic location of the CTX-M gene. The outer ring represents the plasmid replicon detected in each isolate.

A phylogenetic tree based on the core genome (2,459,304 bp for a total of 2,532 genes) was constructed. It showed a main group of 121 isolates from phylogenetic group A (Figure 4 and Supplementary Figure 2). Seven of those, of different geographical origin clustered between isolates from phylogenetic groups D and C. This sub-group contains four isolates from ST5044, one from ST6798, one from ST8130 and a new ST, found respectively, in 7, 3, and 1 instance in the EnteroBase database, all detected in developing countries.

Analysis using fastBAPS and based on the SNP-based phylogenetic tree of isolates from phylogenetic group A only, revealed a high level of diversity with 73 fastBAPS sub-groups identified (Supplementary Figure 3).

A phylogenetic tree was constructed for isolates from each participant site and confirmed the dissemination of genetically diverse isolates independently of their geographical origin (Supplementary Figure 4). No significant clustering of isolates depending on their origin has been observed.

#### Similarity Matrix

**FIGURE 5:**
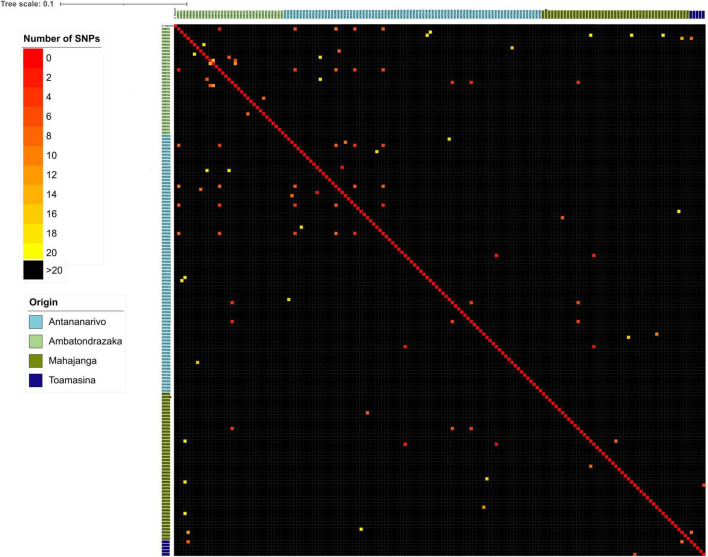
Heat map of SNP differences between ESBL-*E. coli* isolates grouped by geographical origin. Numbers of SNPs in the core genome between different sequenced isolates were interpreted as a distance matrix. Colors in the heat map represent SNP differences as shown in the legend at the top left. The colors of the isolate names indicate their geographical origin.

A similarity matrix based on the core-genome was generated and was used to construct a heat map of isolates differing from each other of up to 20 SNPs. Isolates differing from ≤10 SNPs were explored in more detail (Figure 5). For each sampling location we found a limited number of strains with less than 10 SNPs: 12/82 (14.6%) in Antananarivo, 6/34 (17.7%) in Ambatondrazaka, 2/47 (4.3%) in Mahajanga and none (0/5) in Toamasina. The four couples from Ambatondrazaka differing with ≤10 SNPs belonged to ST7152, ST1716, ST5307 and ST450, the one from Mahajanga belonged to ST1312. In Antananarivo 4 and 2 closely related isolates belonged to ST450, and the three other couples belonged to ST361, ST224 and an unknown ST. All of them were isolated from participants living in different districts of Antananarivo (Supplementary Figure 1).

Some closely related isolates with ≤10 SNPs were also retrieved from different cities, with six ST450 detected in Antananarivo and Ambatondrazaka (2–10 SNPs) and three other ST450 isolates detected in Antananarivo and Mahajanga (2–3 SNPs). The two remaining ST450 isolates from Mahajanga were unrelated to the other ST450 isolates (more than 3000 SNPs difference).

**FIGURE 6:**
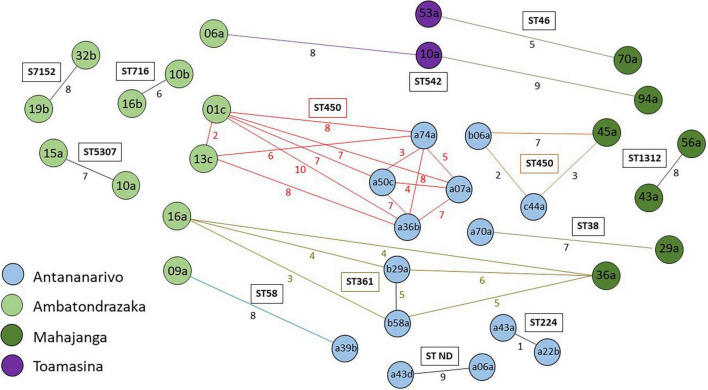
Wireframe diagram showing connections between isolates differing of less than 10 SNPs. The colors of the isolate names indicate their geographical origin. Inter-connections length is independent of the number of SNPs difference between isolates. Numbers on lines indicate the number of SNP differences. Colored lines and numbers indicate distances between closely related isolates from different sampling sites.

Four closely related isolates of ST361 (≤6 SNPs) and three of ST542 (≤9SNPs), were retrieved in three of the four cities: the former in Antananarivo, Mahajanga and Ambatondrazaka and the latter in Mahajanga, Ambatondrazaka and Toamasina. Two other closely related couples were retrieved in two different cities and belonged to ST38 and ST48 (Figure 6).

#### Virulence Genes

**FIGURE 7:**
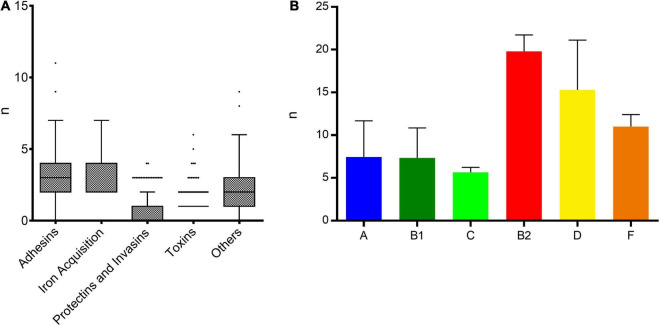
Distribution of virulence genes among ESBL-*E. coli* isolates. **(A)** Distribution of virulence genes per strain among the five main functional classes of virulence. **(B)** Mean number of virulence genes per isolate depending on the phylogenetic group.

Few virulence genes were detected and belonged mostly to pathways involved in adhesion and iron acquisition (Figure 7A). Isolates from commensal phylogenetic groups A, B1, and C carried an average of 7.5, 7.3, and 5.7 virulence genes per isolate while isolates from extra-intestinal virulent phylogenetic groups B2, D, and F carried an average of 19.8, 15.3, and 11 genes, respectively (Figure 7B). The most frequent genes were the adherence factor *fdeC* (82.1%, *n* = 138) and *fimH* (78%, *n* = 131). Toxin genes such as *usp* (uropathogenic-specific protein), *cnf1* (cytotoxic necrotizing factor 1), and *hlyA* (hemolysin A) were detected mainly in isolates from the B2 phylogenetic group and accounted for 3% (*n* = 5), 1.2% (*n* = 2), and 2.4% (*n* = 4), respectively. Isolates belonging to ST450 (phylogenetic group A) shared a highly similar pattern of 7 virulence genes (*fyuA, irp, iucA, iutA, traT, ompT*, and *sat*), (Supplementary Figure 3).

#### Plasmid Analysis

**FIGURE 8:**
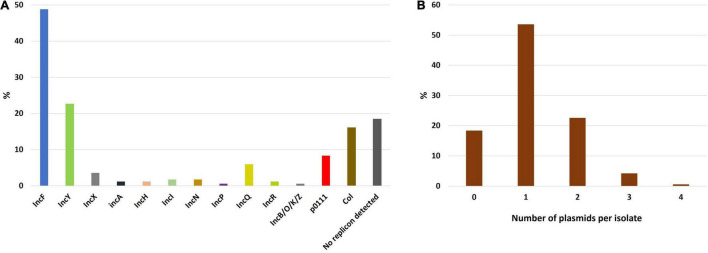
Prevalence of plasmid replicons detected in ESBL-*E. coli* isolates. The sum of the different plasmid replicon prevalence exceeds 100% as some isolates could carry multiple plasmids simultaneously. **(A)** Prevalence of plasmid replicons among the study isolates. **(B)** Histogram depicting the number of plasmids per isolate.

Plasmid replicon analysis revealed a total of 191 replicons in the 168 isolates (1.1 replicons per isolate on average). The most frequent plasmids were from the IncF incompatibility group, found in 49% of the isolates (*n* = 82), followed by IncY in 23% of the isolates (*n* = 38) and Col (16%, *n* = 27) (Figure 8A). Ninety isolates (53.6%) harbored a single plasmid replicon type, whereas two or more plasmid replicons were identified in 47 isolates (28%). No plasmid replicon was found in 31 isolates (18.4%) suggesting a chromosomal location of the ESBL-encoding gene (Figure 8B). Indeed, for 18 of these the CTX-M location was predicted as chromosomic but as plasmidic for the remaining 13.

**FIGURE 9:**
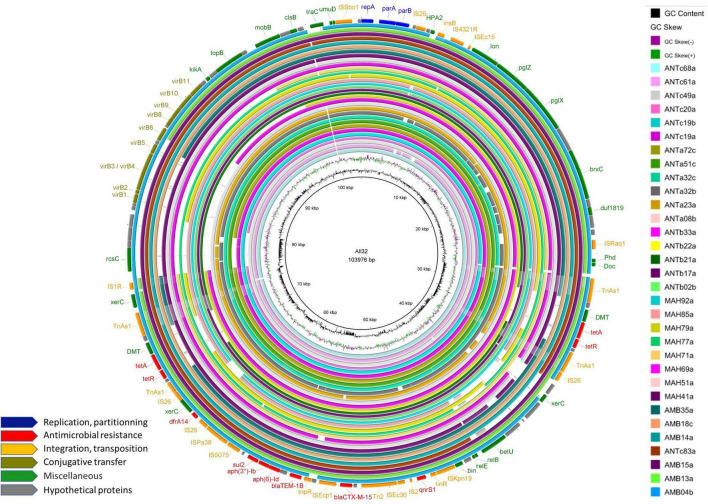
Alignment of the 32 IncY plasmid sequences with sequence homology of more than 85%. GC content and GC skew are depicted in the inner map with distance scale. Predicted coding sequences of the reference plasmid named within the circle are depicted in the outer ring with antimicrobial resistance genes highlighted in red.

We detected IncY plasmid replicons in 23% (*n* = 38) of the ESBL-*E. coli* isolates and decided to perform whole genome sequencing using Nanopore technology in order to rebuild and compare these plasmids. We first compared Illumina contig sequences containing the IncY replicon and *bla*_CTX–M_. We then selected 12 isolates for Nanopore sequencing, 6 with sequence diversity and 6 randomly selected from those remaining with sequence homology. The length of the longest IncY sequence retrieved was 103,976 bp and was used as a reference sequence to align the Nanopore or Illumina contigs of the other isolates with IncY replicons. Of the 38 IncY plasmids detected, 32 showed a high similarity (≥85% identity) (Figure 9). In terms of resistance genes, the reference IncY plasmid carried successively: *tet(A), qnrS1*, *bla*_CTX–M–15_, *bla*_TEM–1B_, *aph(6)-Id, aph(3”)-Ib, sul2, dfrA14* and a second copy of *tet(A)*. In addition, two toxin-antitoxin systems responsible for post-segregational killing were found: the translation inhibition complex Phd-Doc and a mRNA interferase *relE*/*relB*, as well as the lon protease gene responsible for *relE*/*relB* activation. Among other common plasmid genes, we detected partition genes *parA* and *parB* and a *virB* island responsible for conjugation. We did not find any virulence genes carried by these plasmids. Alignment of our IncY reference sequence against GENBANK using BLASTn returned 99.97% sequence identity and 100% coverage with an IncY plasmid from an *E. coli strain* of avian origin isolated in Finland (GenBank accession: LR999865.1).

## Discussion

Thanks to a structured network of laboratories (RESAMAD), we were able to conduct a large-scale study and then report the first results of the human community component of the Tricycle protocol in Madagascar. Our main results highlight a very high ESBL-*E. coli* carriage rate in healthy pregnant women both in the capital and in province with significant variations related to seasonality. We noted a high genomic diversity of strains carrying the *bla*_CTX–M–15_ ESBL gene and belonging mostly to phylogenetic group A.

We chose to conduct the present study in Madagascar because of its specific characteristics. Besides the fact that little is known about ESBL-E in the country, it is a large island in the Indian ocean with a unique ecosystem and an important crossroads between Africa and Asia with significant immigration and commercial exchanges between both continents.

We observed an ESBL-*E. coli* prevalence in pregnant women of 34%, ranging from 12% to 65% depending on the sampling site. Unlike previous studies that focused mainly on the capital Antananarivo and nearby areas, we included three more cities in the provinces to depict a more comprehensive picture of ESBL-E dissemination in the country. Two previous studies conducted in Madagascar, evaluated community carriage of ESBL-E in Antananarivo to 10.1% in 2009 (Herindrainy et al., 2011) and to 18.5% in 2015 in Antananarivo and adjacent areas (Chereau et al., 2015). Our study underlines the worsening problem of ESBL dissemination in Madagascar as ESBL-*E. coli* alone was isolated from 34% of the participants which is much higher than previous reports. These results place Madagascar among the countries with the highest rates of ESBL prevalence confirming the global trends of ESBL epidemiology (Bezabih et al., 2021). This high carriage rate of ESBL-*E. coli* increases the risk of infection caused by ESBL-*E. coli* and, because of probabilistic treatment failures, triggers the use of broad-spectrum beta-lactams such as carbapenems which in its turn drives the spread of carbapenem resistant strains.

The main risk factor for ESBL-*E. coli* carriage that we identified was the wet season, with increased prevalence when rainfall and temperatures are higher. We observed that risk of carriage increased 1.4 times by 100 mm of rainfall and 1.1 times by 1°C of temperature increase. These results are robust with respect to center heterogeneity as shown by the sensitivity analysis with logistic mixed models. Even if this correlation has been described previously in Madagascar (Chereau et al., 2015) as well as in other parts of the world (MacFadden et al., 2018; McGough et al., 2020; Wielders et al., 2020), in light of the large number of scientific reports on ESBL-E carriage, the climate-related risk factor has rarely been reported. This result could be explained by different factors. First the rainy period is well known as being favorable for fecal-oral route of infectious pathogens transmission and enteric bacteria outbreaks (Chowdhury et al., 2018; Chao et al., 2019). In addition, higher prevalence of ESBL-*E. coli* in the rainy and warmer season could further increase horizontal genetic exchanges. It is well established in *in vitro* models that higher growth rates and elevated temperature and humidity are closely related (Ratkowsky et al., 1982) and could promote horizontal gene transfer (Hashimoto et al., 2019). Therefore, one could expect an even worse situation in the future with climate change and global warming trends (Ukuhor, 2021). We found no clear link with socio-economic factors which suggests that ESBL-*E. coli* disseminates evenly in the community in Madagascar mediated by environmental and person to person exchanges. Obviously other factors such as environmental and food contamination may play an important role in the high prevalence of ESBL-E carriage in the human community. It is also important to note that ESBL-E colonizing the gut microbiota of pregnant women can be transmitted to the child during delivery and thus increase the risk of severe ESBL-E infections in newborns (Danino et al., 2018; Herindrainy et al., 2018). Indeed, the incidence rate of severe neonatal infections with multidrug-resistant bacteria (mainly ESBL-E) was estimated at 5 cases per 1,000 live births in three developing countries, including Madagascar. Unfortunately, the origin of the bacteria responsible for the infection (mother, family members, and hospital acquisition) was not investigated (Huynh et al., 2021).

The most frequent ESBL gene identified was by far *bla*_CTX–M–15_ which is consistent with global ESBL epidemiology (Bevan et al., 2017). This is probably due to successful *E. coli* strains carrying *bla*_CTX–M–15_ on IncF plasmids, known for their persistence even in the absence of selective pressure (Mahérault et al., 2019). A high proportion of blaCTX-M genes (32%) was predicted to be chromosomic which is in agreement with previous studies (Hamamoto and Hirai, 2019). Chromosomic location provides stabilization of the blaCTX-M gene without additional cost, unlike a plasmid, and could help maintain resistance in the environment, even without selection pressure (Yoon et al., 2020; Shawa et al., 2021). Given that the protocol of the study was not designed to specifically target carbapenemase producing *E. coli*, isolation of an NDM-5 producing strain suggests a spread of carbapenemase producing *Enterobacterales* in the community of Mahajanga. To our knowledge, this is the first description of NDM-5 metalo beta-lactamase in Madagascar. Carbapenems are available in the country but their use is limited because of their high price (Herindrainy et al., 2011). Therefore, isolation of a strain carrying *bla*_*NDM–*5_ in the absence of selective pressure is extremely worrying.

The molecular analysis of the isolates revealed a great variety of strains in all the sampling locations which was also observed in other developing countries (Stoesser et al., 2015; Ramadan et al., 2020; Foster-Nyarko et al., 2021). The majority were from commensal phylogenetic groups A with STc10 being the most frequent clonal complex detected. The second most frequent was STc155 (also named CC87 using the Pasteur Institute MLST scheme) with isolates from this STc belonging mainly to ST58 and ST155. STc155 strains are considered of animal origin with an evolution from wild type strains, becoming progressively resistant and, in many cases, later acquiring virulence genes (Skurnik et al., 2016). Indeed, in recent years multi-drug resistant (MDR) STc155 strains have been isolated with increasing frequency from extra-intestinal infections in humans (Royer et al., 2021). In our study STc155 isolates carried only a limited number of virulence genes but had already acquired an MDR profile. This finding underlines the importance of animal to human transmission in the dissemination of ESBL-*E. coli* in the community. Our results are consistent with previous reports describing higher prevalence of phylogenetic group A isolates in developing countries in contrast to industrialized countries where phylogenetic group B2 isolates are the most frequent (Duriez et al., 2001; Tenaillon et al., 2010). The phylogenetic tree based on a core genome SNP analysis showed a classical distribution of isolates by phylogenetic group except for a subgroup of phylogenetic group A isolates clustering between isolates from phylogenetic groups D and C. This subgroup contains STs found in very limited numbers in the EnteroBase database, which however, includes over 184,000 *E. coli* genomes. This indicates the potential emergence of a distinct sub-group beginning to diverge genetically from phylogenetic group A. As expected, we found few virulence genes belonging mainly to the adhesins group and a small number of toxins which is consistent with the commensal nature of most of the isolates. Even though most isolates belonged to commensal phylogenetic groups and therefore are less likely to cause invasive disease, infections with commensal strains are not uncommon (Leverstein-van Hall et al., 2011; Dadi et al., 2020). Also, one can imagine that these ESBL-*E. coli* strains can reside for a long time in the gut microbiota as they are well adapted to their host and thus represent an significant reservoir of resistance genes and a potential source that drives human to human transmission (Tande et al., 2010).

To analyze more precisely ESBL-*E. coli* dissemination we focused on isolates differing by less than 10 SNPs, a cut-off used in the literature to define closely related isolates (Schürch et al., 2018). Core-genome based SNP analysis revealed a limited number of closely related isolates circulating in each sampling location, which indicates probable inter-human transmission or a common contamination origin. Isolates differing from each other by less than 10 SNPs, especially from ST450 and ST361 have also been observed in different locations. In addition, most inter-city interactions were linked to the capital Antananarivo and to a lesser extent to provincial cities only. Assuming a mutation rate of 1.1 SNP per genome and per year as described elsewhere (Reeves et al., 2011) and equal mutation rates for all isolates, this finding suggests recent inter-city dissemination events probably due to traveling with journeys to the capital playing a central role in dissemination. Another hypothesis is that some clones, may have lower natural mutation rates than others.

Plasmid content analysis revealed an unusually prevalent IncY plasmid compared to other studies (Lyimo et al., 2016; Salinas et al., 2019), suggesting a dissemination in the country of an IncY type plasmid harboring a resistance genes island containing *bla*_CTX–M–15_ and six other resistant genes of different antibiotic classes. To our knowledge, this is the first report of such a high prevalence of an IncY plasmid in ESBL-*E. coli* isolates. The extremely high sequence identity shared with an IncY plasmid in an *E. coli* strain isolated in Finland from *Branta leucopsis* (Kurittu et al., 2021) indicates that migratory birds could represent a powerful means of resistance spread even if a direct link to *E. coli* isolates from Madagascar is unlikely, due to the Northern Hemisphere habitat of this bird species (Owen and Black, 1989).

The present study has several limitations. First, due to logistic constraints, the sampling in the different hospitals was not performed during the same period. This could introduce a bias as risk factors may differ in different seasons. However, we performed a sensitivity analysis with logistic mixed models that addresses this bias. Second, several samples were eliminated because of transportation issues, but we were still able to perform phenotypic and genotypic analysis on 92% of expected isolates. Third, the Tricycle protocol requires three isolates per pregnant woman but certain participant hospitals did not collect all the three. Moreover, in some cases less than three presumptive ESBL-*E. coli* colonies were present after sample culture. In all, we were able to obtain three isolates in 42% of the participants. Moreover, even when successfully obtained, these three isolates often represent the dominant colonizing strains colonizing and one cannot exclude the existence of extensive in-host genetic diversity among non-dominant bacterial populations. Finally, we were able to sequence by the Nanopore technology only 12 out of 38 isolates carrying IncY plasmid due to budget limitations but we tried to compensate for this by aligning the Illumina reads of the remaining isolates to the IncY sequences reconstructed from the Nanopore sequencing.

## Conclusion

We report here that in 2018–2019 more than one third of pregnant women in four study cities in Madagascar were ESBL-*E. coli* carriers, which probably reflects high carriage of ESBL-*E. coli* in the community. As the warmer and rainy season was the main risk factor associated with ESBL-*E. coli* carriage, it is reasonable to assume that the situation could grow even worse with climate change trends. Continued surveillance efforts should be maintained along with public health interventions to, if not reverse, at least slow down the spread of resistance in Madagascar. Our results will have to be reconsidered in a One Health context by linking them to the results of the ESBL *E. coli* prevalence in the food chain and the environment which is the next step of the Tricycle protocol to better understand the complex face of the antimicrobial resistance.

## Data Availability Statement

The original contributions presented in the study are publicly available. These data can be found here: https://www.ebi.ac.uk/ena/browser/home (accession number PRJEB48998).

## Ethics Statement

The studies involving human participants were reviewed and approved by the Ethics Committee of Ministry of Health, Madagascar. Written informed consent to participate in this study was provided by the participants’ legal guardian/next of kin.

## Author Contributions

MM carried out the experiments, analyzed the results, and wrote the first draft of the manuscript. SR, LVR, RR, RC, CR, ER, and ZR collected the samples and data, and performed the experiments in Madagascar. EW and MP performed the bioinformatic analyses. OC participated in the experiments and analysis of the WGS results. JM performed the statistical analyses. LR, LS, and HE were involved in the implementation of the project in Madagascar and supervised its progress. FK-P managed the implementation of the project and its conduct, and participated in the analysis of the results. AA was involved in the original idea of Tricycle and design of the study. ED was involved in the analysis of the WGS data and revision of the manuscript. LA-L was responsible for the overall direction and planning, supervision of all experiments and results, and writing the manuscript. All authors contributed to the revision of the manuscript and read and approved the submitted version.

## Conflict of Interest

The authors declare that the research was conducted in the absence of any commercial or financial relationships that could be construed as a potential conflict of interest.

## Publisher’s Note

All claims expressed in this article are solely those of the authors and do not necessarily represent those of their affiliated organizations, or those of the publisher, the editors and the reviewers. Any product that may be evaluated in this article, or claim that may be made by its manufacturer, is not guaranteed or endorsed by the publisher.

## Footnotes

1
https://www.genomicepidemiology.org/

2
https://enterobase.warwick.ac.uk/species/index/ecoli

## References

[B1] Alikhan N.-F. Petty N. K. Ben Zakour N. L. Beatson S. A. (2011). BLAST ring image generator (BRIG): simple prokaryote genome comparisons. *BMC Genomics* 12:402. 10.1186/1471-2164-12-402 21824423 PMC3163573

[B2] Beghain J. Bridier-Nahmias A. Le Nagard H. Denamur E. Clermont O. (2018). ClermonTyping: an easy-to-use and accurate in silico method for *Escherichia* genus strain phylotyping. *Microb. Genomics* 4:e000192. 10.1099/mgen.0.000192 29916797 PMC6113867

[B3] Bevan E. R. Jones A. M. Hawkey P. M. (2017). Global epidemiology of CTX-M β-lactamases: temporal and geographical shifts in genotype. *J. Antimicrob. Chemother.* 72 2145–2155. 10.1093/jac/dkx146 28541467

[B4] Bezabih Y. M. Sabiiti W. Alamneh E. Bezabih A. Peterson G. M. Bezabhe W. M. (2021). The global prevalence and trend of human intestinal carriage of ESBL-producing *Escherichia coli* in the community. *J. Antimicrob. Chemother.* 76 22–29. 10.1093/jac/dkaa399 33305801

[B5] Bourrel A. S. Poirel L. Royer G. Darty M. Vuillemin X. Kieffer N. (2019). Colistin resistance in Parisian inpatient faecal *Escherichia coli* as the result of two distinct evolutionary pathways. *J. Antimicrob. Chemother.* 74 1521–1530. 10.1093/jac/dkz090 30863849

[B6] Carattoli A. Zankari E. García-Fernández A. Voldby Larsen M. Lund O. Villa L. (2014). In silico detection and typing of plasmids using plasmidfinder and plasmid multilocus sequence typing. *Antimicrob. Agents Chemother.* 58 3895–3903. 10.1128/AAC.02412-14 24777092 PMC4068535

[B7] Chao D. L. Roose A. Roh M. Kotloff K. L. Proctor J. L. (2019). The seasonality of diarrheal pathogens: a retrospective study of seven sites over three years. *PLoS Negl. Trop. Dis.* 13:e0007211. 10.1371/journal.pntd.0007211 31415558 PMC6711541

[B8] Chereau F. Herindrainy P. Garin B. Huynh B.-T. Randrianirina F. Padget M. (2015). Colonization of extended-spectrum-β-lactamase- and NDM-1-producing *Enterobacteriaceae* among pregnant women in the community in a low-income country: a potential reservoir for transmission of multiresistant *Enterobacteriaceae* to neonates. *Antimicrob. Agents Chemother.* 59 3652–3655. 10.1128/AAC.00029-15 25845871 PMC4432137

[B9] Chowdhury F. R. Ibrahim Q. S. U. Bari M. D. S. Alam M. M. J. Dunachie S. J. Rodriguez-Morales A. J. (2018). The association between temperature, rainfall and humidity with common climate-sensitive infectious diseases in Bangladesh. *PLoS One* 13:e0199579. 10.1371/journal.pone.0199579 29928056 PMC6013221

[B10] Dadi B. R. Abebe T. Zhang L. Mihret A. Abebe W. Amogne W. (2020). Distribution of virulence genes and phylogenetics of uropathogenic *Escherichia coli* among urinary tract infection patients in Addis Ababa, Ethiopia. *BMC Infect. Dis.* 20:108. 10.1186/s12879-020-4844-z 32033541 PMC7006406

[B11] Danino D. Melamed R. Sterer B. Porat N. Hazan G. Gushanski A. (2018). Mother-to-child transmission of extended-spectrum-beta-lactamase-producing Enterobacteriaceae. *J. Hosp. Infect.* 100, 40–46. 10.1016/j.jhin.2017.12.024 29330015

[B12] Duriez P. Clermont O. Bonacorsi S. Bingen E. Chaventré A. Elion J. (2001). Commensal *Escherichia coli* isolates are phylogenetically distributed among geographically distinct human populations. *Microbiology* 147 1671–1676. 10.1099/00221287-147-6-1671 11390698

[B13] European Committee on Antimicrobial Susceptibility Testing, Clinical breakpoints (2019). *European Committee on Antimicrobial Susceptibility Testing (2018) Clinical breakpoints.* Available: https://www.eucast.org/clinical_breakpoints (accessed November 2018).

[B14] Foster-Nyarko E. Alikhan N.-F. Ikumapayi U. N. Sarwar G. Okoi C. Tientcheu P.-E. M. (2021). Genomic diversity of *Escherichia coli* from healthy children in rural Gambia. *PeerJ* 9:e10572. 10.7717/peerj.10572 33505796 PMC7796664

[B15] Guindon S. Dufayard J.-F. Lefort V. Anisimova M. Hordijk W. Gascuel O. (2010). New algorithms and methods to estimate maximum-likelihood phylogenies: assessing the performance of PhyML 3.0. *Syst. Biol.* 59 307–321. 10.1093/sysbio/syq010 20525638

[B16] Hamamoto K. Hirai I. (2019). Characterisation of chromosomally-located blaCTX-M and its surrounding sequence in CTX-M-type extended-spectrum β-lactamase-producing *Escherichia coli* isolates. *J. Glob. Antimicrob. Resist.* 17 53–57. 10.1016/j.jgar.2018.11.006 30445208

[B17] Hashimoto M. Hasegawa H. Maeda S. (2019). High temperatures promote cell-to-cell plasmid transformation in *Escherichia coli*. *Biochem. Biophys. Res. Commun.* 515 196–200. 10.1016/j.bbrc.2019.05.134 31138439

[B18] Herindrainy P. Randrianirina F. Ratovoson R. Ratsima Hariniana E. Buisson Y. Genel N. (2011). Rectal carriage of extended-spectrum beta-lactamase-producing gram-negative bacilli in community settings in madagascar. *PLoS One* 6:e22738. 10.1371/journal.pone.0022738 21829498 PMC3146483

[B19] Herindrainy P. Rabenandrasana M. A. N. Andrianirina Z. Z. Rakotoarimanana F. M. J. Padget M. de Lauzanne A. (2018). Acquisition of extended spectrum beta-lactamase-producing Enterobacteriaceae in neonates: a community based cohort in Madagascar. *PLoS One* 13:e0193325. 10.1371/journal.pone.0193325 29494706 PMC5832238

[B20] Huynh B.-T. Kermorvant-Duchemin E. Chheang R. Randrianirina F. Seck A. Hariniaina Ratsima E. (2021). Severe bacterial neonatal infections in Madagascar, Senegal, and Cambodia: a multicentric community-based cohort study. *PLoS Med.* 18:e1003681. 10.1371/journal.pmed.1003681 34582450 PMC8478182

[B21] Joensen K. G. Scheutz F. Lund O. Hasman H. Kaas R. S. Nielsen E. M. (2014). Real-time whole-genome sequencing for routine typing, surveillance, and outbreak detection of verotoxigenic Escherichia coli. *J. Clin. Microbiol.* 52, 1501–1510. 10.1128/JCM.03617-13 24574290 PMC3993690

[B22] Joensen K. G. Tetzschner A. M. M. Iguchi A. Aarestrup F. M. Scheutz F. (2015). Rapid and easy in silico serotyping of *Escherichia coli* isolates by use of whole-genome sequencing data. *J. Clin. Microbiol.* 53 2410–2426. 10.1128/JCM.00008-15 25972421 PMC4508402

[B23] Karanika S. Karantanos T. Arvanitis M. Grigoras C. Mylonakis E. (2016). Fecal colonization with extended-spectrum beta-lactamase–producing *Enterobacteriaceae* and risk factors among healthy individuals: a systematic review and metaanalysis. *Clin. Infect. Dis.* 63 310–318. 10.1093/cid/ciw283 27143671

[B24] Kurittu P. Khakipoor B. Brouwer M. S. M. Heikinheimo A. (2021). Plasmids conferring resistance to extended-spectrum beta-lactamases including a rare IncN+IncR multireplicon carrying blaCTX-M-1 in *Escherichia coli* recovered from migrating barnacle geese (*Branta leucopsis*). *Open Res. Eur.* 1:46. 10.12688/openreseurope.13529.1 PMC10446048 37645149

[B25] Larsen M. V. Cosentino S. Rasmussen S. Friis C. Hasman H. Marvig R. L. (2012). Multilocus sequence typing of total-genome-sequenced bacteria. *J. Clin. Microbiol.* 50 1355–1361. 10.1128/JCM.06094-11 22238442 PMC3318499

[B26] Letunic I. Bork P. (2007). Interactive Tree Of Life (iTOL): an online tool for phylogenetic tree display and annotation. *Bioinformatics* 23 127–128. 10.1093/bioinformatics/btl529 17050570

[B27] Leverstein-van Hall M. A. Dierikx C. M. Stuart J. C. Voets G. M. van den Munckhof M. P. van Essen-Zandbergen A. (2011). Dutch patients, retail chicken meat and poultry share the same ESBL genes, plasmids and strains. *Clin. Microbiol. Infect.* 17 873–880. 10.1111/j.1469-0691.2011.03497.x 21463397

[B28] Liu B. Zheng D. Jin Q. Chen L. Yang J. (2019). VFDB 2019: a comparative pathogenomic platform with an interactive web interface. *Nucleic Acids Res.* 47 D687–D692. 10.1093/nar/gky1080 30395255 PMC6324032

[B29] Lyimo B. Buza J. Subbiah M. Temba S. Kipasika H. Smith W. (2016). IncF plasmids are commonly carried by antibiotic resistant *Escherichia coli* isolated from drinking water sources in Northern Tanzania. *Int. J. Microbiol.* 2016 1–7. 10.1155/2016/3103672 27110245 PMC4823495

[B30] Lynch J. P. Clark N. M. Zhanel G. G. (2013). Evolution of antimicrobial resistance among *Enterobacteriaceae* (focus on extended spectrum β-lactamases and carbapenemases). *Expert Opin. Pharmacother.* 14 199–210. 10.1517/14656566.2013.763030 23321047

[B31] MacFadden D. R. McGough S. F. Fisman D. Santillana M. Brownstein J. S. (2018). Antibiotic resistance increases with local temperature. *Nat. Clim. Change* 8 510–514. 10.1038/s41558-018-0161-6 30369964 PMC6201249

[B32] Mahérault A.-C. Kemble H. Magnan M. Gachet B. Roche D. Le Nagard H. (2019). Advantage of the F2:A1:B- IncF pandemic plasmid over IncC plasmids in in vitro acquisition and evolution of bla CTX-M gene-bearing plasmids in *Escherichia coli*. *Antimicrob. Agents Chemother.* 63:e01130-19. 10.1128/AAC.01130-19 31332067 PMC6761558

[B33] McGough S. F. MacFadden D. R. Hattab M. W. Mølbak K. Santillana M. (2020). Rates of increase of antibiotic resistance and ambient temperature in Europe: a cross-national analysis of 28 countries between 2000 and 2016. *Eurosurveillance* 25:1900414. 10.2807/1560-7917.ES.2020.25.45.1900414 33183408 PMC7667635

[B34] Owen M. Black J. M. (1989). Factors affecting the survival of barnacle geese on migration from the breeding grounds. *J. Anim. Ecol.* 58:603. 10.2307/4851

[B35] Page A. J. Cummins C. A. Hunt M. Wong V. K. Reuter S. Holden M. T. G. (2015). Roary: rapid large-scale prokaryote pan genome analysis. *Bioinformatics* 31 3691–3693. 10.1093/bioinformatics/btv421 26198102 PMC4817141

[B36] Ramadan H. Jackson C. R. Frye J. G. Hiott L. M. Samir M. Awad A. (2020). Antimicrobial resistance, genetic diversity and multilocus sequence typing of *Escherichia coli* from humans, retail chicken and ground beef in Egypt. *Pathogens* 9:357. 10.3390/pathogens9050357 32397188 PMC7281645

[B37] Ratkowsky D. A. Olley J. McMeekin T. A. Ball A. (1982). Relationship between temperature and growth rate of bacterial cultures. *J. Bacteriol.* 149 1–5. 10.1128/JB.149.1.1-5.1982 7054139 PMC216584

[B38] Reeves P. R. Liu B. Zhou Z. Li D. Guo D. Ren Y. (2011). Rates of mutation and host transmission for an *Escherichia coli* clone over 3 years. *PLoS One* 6:e26907. 10.1371/journal.pone.0026907 22046404 PMC3203180

[B39] Royer G. Darty M. M. Clermont O. Condamine B. Laouenan C. Decousser J.-W. (2021). Phylogroup stability contrasts with high within sequence type complex dynamics of *Escherichia coli* bloodstream infection isolates over a 12-year period. *Genome Med.* 13:77. 10.1186/s13073-021-00892-0 33952335 PMC8097792

[B40] Royer G. Decousser J. W. Branger C. Dubois M. Médigue C. Denamur E. (2018). PlaScope: a targeted approach to assess the plasmidome from genome assemblies at the species level. *Microb. Genomics* 4:e000211. 10.1099/mgen.0.000211 30265232 PMC6202455

[B41] Salinas L. Cárdenas P. Johnson T. J. Vasco K. Graham J. Trueba G. (2019). Diverse commensal *E. coli* clones and plasmids disseminate antimicrobial resistance genes in domestic animals and children in a semi-rural community in Ecuador. *Microbiology* 4:e00316-19. 10.1128/mSphere.00316-19 PMC6531886 31118304

[B42] Savageau M. A. (1983). *Escherichia coli* habitats, cell types, and molecular mechanisms of gene control. *Am. Nat.* 122 732–744. 10.1086/284168

[B43] Schürch A. C. Arredondo-Alonso S. Willems R. J. L. Goering R. V. (2018). Whole genome sequencing options for bacterial strain typing and epidemiologic analysis based on single nucleotide polymorphism versus gene-by-gene–based approaches. *Clin. Microbiol. Infect.* 24 350–354. 10.1016/j.cmi.2017.12.016 29309930

[B44] Schwaber M. J. Carmeli Y. (2007). Mortality and delay in effective therapy associated with extended-spectrum -lactamase production in *Enterobacteriaceae* bacteraemia: a systematic review and meta-analysis. *J. Antimicrob. Chemother.* 60 913–920. 10.1093/jac/dkm318 17848376

[B45] Seemann T. (2014). Prokka: rapid prokaryotic genome annotation. *Bioinform. Oxf. Engl.* 30 2068–2069. 10.1093/bioinformatics/btu153 24642063

[B46] Shawa M. Furuta Y. Mulenga G. Mubanga M. Mulenga E. Zorigt T. (2021). Novel chromosomal insertions of ISEcp1-blaCTX-M-15 and diverse antimicrobial resistance genes in Zambian clinical isolates of *Enterobacter cloacae* and *Escherichia coli*. *Antimicrob. Resist. Infect. Control* 10:79. 10.1186/s13756-021-00941-8 33971966 PMC8111917

[B47] Skurnik D. Clermont O. Guillard T. Launay A. Danilchanka O. Pons S. (2016). Emergence of antimicrobial-resistant *Escherichia coli* of animal origin spreading in humans. *Mol. Biol. Evol.* 33 898–914. 10.1093/molbev/msv280 26613786 PMC5013867

[B48] Stoesser N. Sheppard A. E. Moore C. E. Golubchik T. Parry C. M. Nget P. (2015). Extensive within-host diversity in fecally carried extended-spectrum-beta-lactamase-producing *Escherichia coli* isolates: implications for transmission analyses. *J. Clin. Microbiol.* 53 2122–2131. 10.1128/JCM.00378-15 25903575 PMC4473215

[B49] Tande D. Boisrame-Gastrin S. Munck M. R. Hery-Arnaud G. Gouriou S. Jallot N. (2010). Intrafamilial transmission of extended-spectrum- -lactamase-producing *Escherichia coli* and *Salmonella enterica* Babelsberg among the families of internationally adopted children. *J. Antimicrob. Chemother.* 65 859–865. 10.1093/jac/dkq068 20233775

[B50] Tenaillon O. Skurnik D. Picard B. Denamur E. (2010). The population genetics of commensal *Escherichia coli*. *Nat. Rev. Microbiol.* 8 207–217. 10.1038/nrmicro2298 20157339

[B51] Treangen T. J. Ondov B. D. Koren S. Phillippy A. M. (2014). The Harvest suite for rapid core-genome alignment and visualization of thousands of intraspecific microbial genomes. *Genome Biol.* 15:524. 10.1186/s13059-014-0524-x 25410596 PMC4262987

[B52] Ukuhor H. O. (2021). The interrelationships between antimicrobial resistance, COVID-19, past, and future pandemics. *J. Infect. Public Health* 14 53–60. 10.1016/j.jiph.2020.10.018 33341485 PMC7831651

[B53] Wick R. R. Judd L. M. Gorrie C. L. Holt K. E. (2017). Unicycler: Resolving bacterial genome assemblies from short and long sequencing reads. *PLoS Comput. Biol.* 13:e1005595. 10.1371/journal.pcbi.1005595 28594827 PMC5481147

[B54] Wielders C. C. H. Van Duijkeren E. Van Den Bunt G. Meijs A. P. Dierikx C. M. Bonten M. J. M. (2020). Seasonality in carriage of extended-spectrum β -lactamase-producing *Escherichia coli* and *Klebsiella pneumoniae* in the general population: a pooled analysis of nationwide cross-sectional studies. *Epidemiol. Infect.* 148:e68. 10.1017/S0950268820000539 32081112 PMC7118714

[B55] Woerther P.-L. Angebault C. Jacquier H. Hugede H.-C. Janssens A.-C. Sayadi S. (2011). Massive increase, spread, and exchange of extended spectrum -lactamase-encoding genes among intestinal *Enterobacteriaceae* in hospitalized children with severe acute malnutrition in niger. *Clin. Infect. Dis.* 53 677–685. 10.1093/cid/cir522 21890771

[B56] Woerther P.-L. Burdet C. Chachaty E. Andremont A. (2013). Trends in human fecal carriage of extended-spectrum -lactamases in the community: toward the globalization of CTX-M. *Clin. Microbiol. Rev.* 26 744–758. 10.1128/CMR.00023-13 24092853 PMC3811232

[B57] World Health Organization [WHO] (2015). *Global Action Plan on Antimicrobial Resistance.* Geneva: WHO.

[B58] World Health Organization [WHO] (2021). *WHO Integrated Global Surveillance on ESBL-Producing E. coli Using a “One Health” Approach.* Geneva: WHO.

[B59] Yoon E.-J. Gwon B. Liu C. Kim D. Won D. Park S. G. (2020). Beneficial chromosomal integration of the genes for CTX-M extended-spectrum β-Lactamase in *Klebsiella pneumoniae* for stable propagation. *mSystems* 5:e00459-20. 10.1128/mSystems.00459-20 32994286 PMC7527135

[B60] Zankari E. Hasman H. Cosentino S. Vestergaard M. Rasmussen S. Lund O. (2012). Identification of acquired antimicrobial resistance genes. *J. Antimicrob. Chemother.* 67 2640–2644. 10.1093/jac/dks261 22782487 PMC3468078

[B61] Zhou Z. Alikhan N.-F. Sergeant M. J. Luhmann N. Vaz C. Francisco A. P. (2018). GrapeTree: visualization of core genomic relationships among 100,000 bacterial pathogens. *Genome Res.* 28 1395–1404. 10.1101/gr.232397.117 30049790 PMC6120633

