# Supplementary material for: Prevalence, Risk Factors, and Genetic Characterization of Extended-Spectrum Beta-Lactamase Escherichia coli Isolated From Healthy Pregnant Women in Madagascar
Source: Front Microbiol. 2021 Dec 24;12:786146. doi: 10.3389/fmicb.2021.786146 (PMC8740230; doi:10.3389/fmicb.2021.786146)
Supplement: Supplementary file 2 [file Data_Sheet_2.PDF]

Figure S2. Phylogenetic tree based on the core genome sequences of all isolates. The tree has been constructed with *E. fergusonii* used as reference. Isolates names are highlighted depending on their phylogenetic group (first strip). Strip two represents the geographical origin of the isolates. ST and STc are given in the third and fourth strips. Serotypes and fimH types are depicted in strip five and six respectively. The CTX-M enzyme detected is presented in strip seven and its genomic location in strip eight. The penultimate strip represents the plasmid replicon detected in each isolate. Virulence genes are depicted in the last strip.

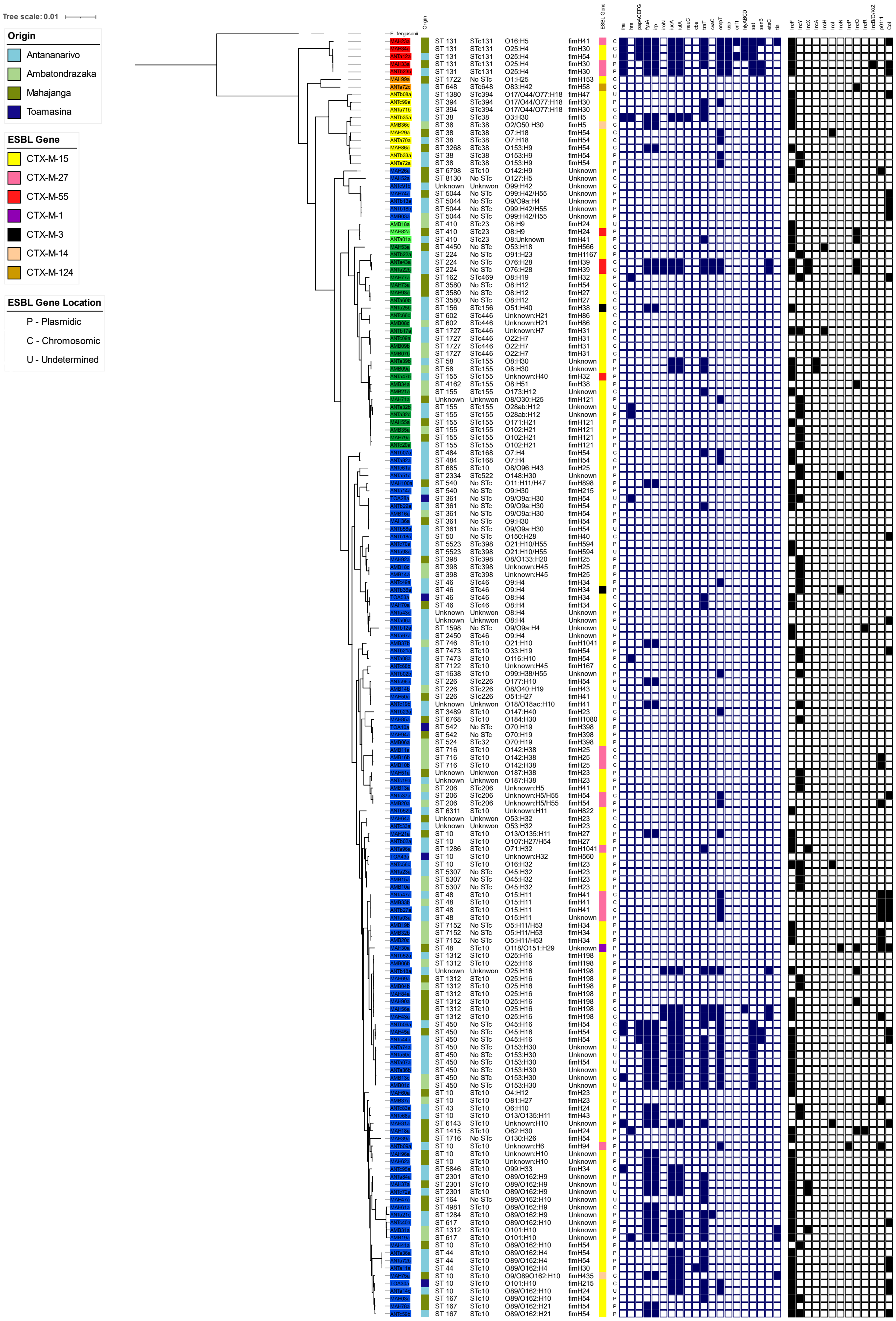

Tree scale: 0.01
Origin
Antananarivo
Ambatondrazaka
Mahajanga
Toamasina
ESBL Gene
CTX-M-15
CTX-M-27
CTX-M-55
CTX-M-1
CTX-M-3
CTX-M-14
CTX-M-124
ESBL Gene Location
P - Plasmidic
C - Chromosomic
U - Undetermined
E. fergusonii
Origin
ESBL Gene
