# Supplementary material for: Prevalence, Risk Factors, and Genetic Characterization of Extended-Spectrum Beta-Lactamase Escherichia coli Isolated From Healthy Pregnant Women in Madagascar
Source: Front Microbiol. 2021 Dec 24;12:786146. doi: 10.3389/fmicb.2021.786146 (PMC8740230; doi:10.3389/fmicb.2021.786146)

Figure S3. Phylogenetic tree of phylogenetic group A isolates. The tree has been constructed with an *E. coli* strain of phylogenetic group *B1* as reference. Strip one represents fastBAPS groups. Coloured strips represent the geographical origin of the isolates (strip two) and the CTX-M enzyme detected (strip six). STs, Serotypes and FimH are given in the third, fourth and fifth strips respectively. The seventh strip represents the genomic location of the CTX-M gene. The three last strips depict virulence genes, plasmid content and fluoroquinolone resistance mutations respectively.

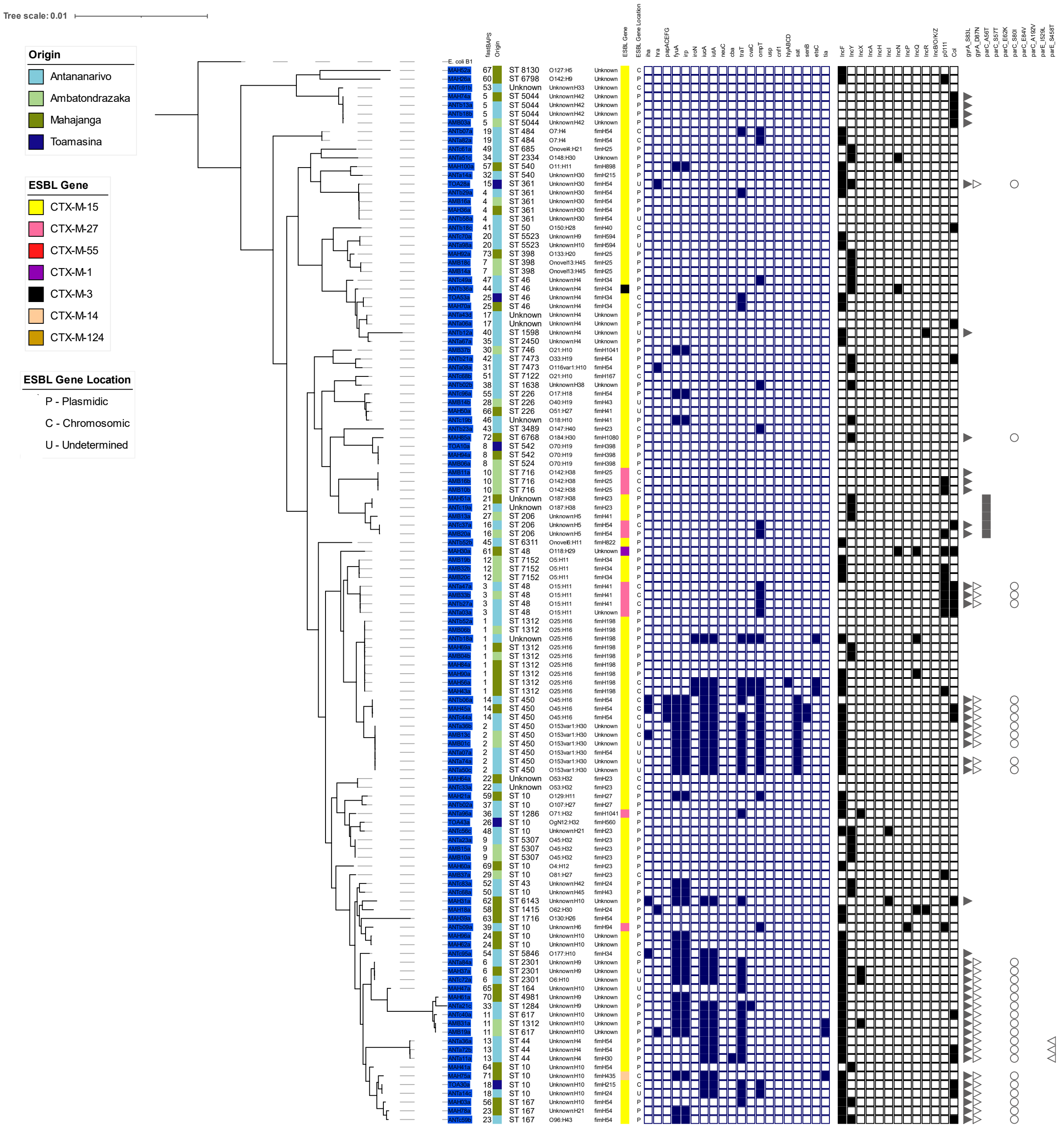

Supplement: Supplementary file 3 [file Data_Sheet_3.PDF]
