# Supplementary material for: Prevalence, Risk Factors, and Genetic Characterization of Extended-Spectrum Beta-Lactamase Escherichia coli Isolated From Healthy Pregnant Women in Madagascar
Source: Front Microbiol. 2021 Dec 24;12:786146. doi: 10.3389/fmicb.2021.786146 (PMC8740230; doi:10.3389/fmicb.2021.786146)

Figure S4. Phylogenetic tree based on core genome sequences of isolates depending on their origin. The trees have been constructed with *E. fergusonii* used as reference. Isolates names are highlighted depending on their phylogenetic group (inner ring). **A : Ambatondrazaka, B : Mahajanga D : Toamasina** - Coloured strip two represent the CTX-M enzyme detected. The third strip represents the genomic location of the CTX-M gene. The outer strip represents the plasmid replicon detected in each isolate. **C : Antananarivo** – The second strip represents the different sampling sites in the capital (JRA - Joseph Ravoahangy Andrianavalona hospital, JRB – Joseph Raseta Befelatanana hospital, TSA - Mère-Enfant Tsaralalana hospital).

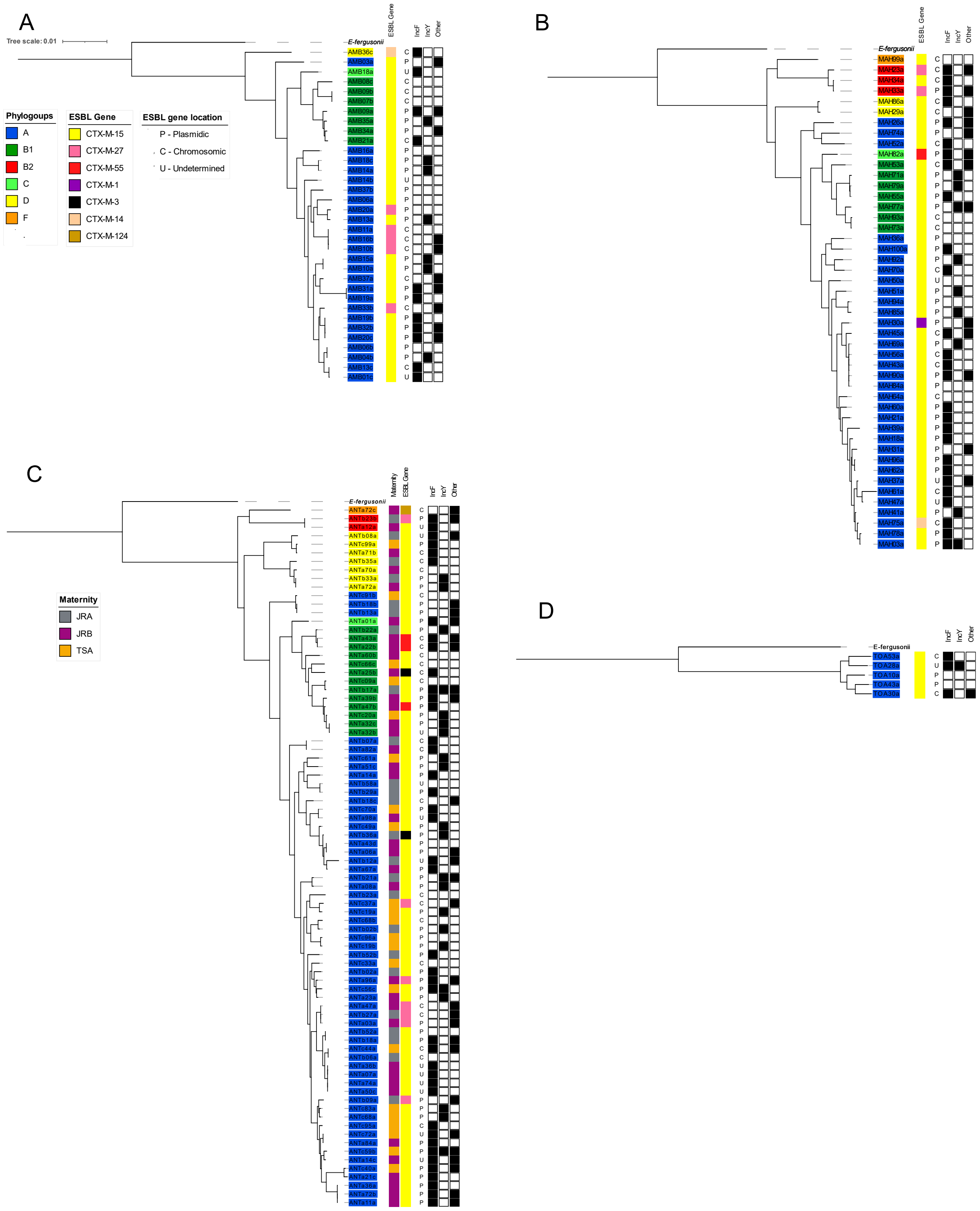

Supplement: Supplementary file 4 [file Data_Sheet_4.PDF]
