# Supplementary material for: Prevalence, Risk Factors, and Genetic Characterization of Extended-Spectrum Beta-Lactamase Escherichia coli Isolated From Healthy Pregnant Women in Madagascar
Source: Front Microbiol. 2021 Dec 24;12:786146. doi: 10.3389/fmicb.2021.786146 (PMC8740230; doi:10.3389/fmicb.2021.786146)
Supplement: Supplementary file 5 [file Table_1.DOCX]

**Table S1. Antibiotic susceptibility of ESBL-*E. coli* isolates**

R: resistant, I: intermediate susceptibility, S: susceptible, / : Intermediate susceptibility not defined by EUCAST guidelines

| **Antibiotic** | **ESBL-*E. coli* isolates (n=168)** | | |
| --- | --- | --- | --- |
|  | **R** | **I** | **R + I** |
| Amoxicillin/Clavulanic Acid | 23 (13.7) | / | 23 (13.7) |
| Piperacillin/Tazobactam | 14 (8.3) | 6 (3.6) | 20 (11.9) |
| Cefotaxime | 168 (100) | 0 (0) | 168 (100) |
| Ceftazidime | 105 (62.5) | 37 (22.0) | 142 (84.5) |
| Cefepime | 124 (73.8) | 44 (26.2) | 168 (100) |
| Cefoxitin | 5 (3.0) | 4 (2.4) | 9 (5.4) |
| Temocillin | 19 (11.3) | / | 19 (11.3) |
| Imipenem | 1 (0.6) | 0 (0) | 1 (0.6) |
| Meropenem | 1 (0.6) | 0 (0) | 1 (0.6) |
| Ertapenem | 1 (0.6) | 1 (0.6) | 2 (1.2) |
| Fosfomycin | 1 (0.6) | / | 1 (0.6) |
| Chloramphenicol | 7 (4.2) | / | 7 (4.2) |
| Ofloxacin | 139 (82.7) | 18 (10.7) | 157 (93.5) |
| Ciprofloxacin | 87 (51.8) | 50 (29.8) | 137 (81.5) |
| Amikacin | 0 (0) | 10 (6.0) | 10 (6.0) |
| Gentamicin | 16 (9.5) | 27 (16.1) | 43 (25.6) |
| Tetracycline | 120 (71.4) | 0 (0) | 120 (71.4) |
| Trimethoprim/Sulfamethoxazole | 118 (70.3) | 1 (0.6) | 119 (70.8) |
