# Supplementary material for: Prevalence, Risk Factors, and Genetic Characterization of Extended-Spectrum Beta-Lactamase Escherichia coli Isolated From Healthy Pregnant Women in Madagascar
Source: Front Microbiol. 2021 Dec 24;12:786146. doi: 10.3389/fmicb.2021.786146 (PMC8740230; doi:10.3389/fmicb.2021.786146)
Supplement: Supplementary file 6 [file Table_2.DOCX]

**Table S2. Resistance to Fluoroquinolones**

OFX – Ofloxacine, CIP – Ciprofloxacine, LVX – Levofloxacine; R – Resistant, I – Intermediate, S- Susceptible

| Isolate | Suscepti-bility phenotype | | | Fluoro-quinolone resistance gene | Point mutations | | | | | | | | | |
| --- | --- | --- | --- | --- | --- | --- | --- | --- | --- | --- | --- | --- | --- | --- |
|  |  |  |  |  | *gyrA* | | *parC* | | | | | | *parE* | |
|  | OFX | CIP | LVX |  | S83L | D87N | A56T | S57T | E62K | S80I | E84V | A192V | S458T | I529L |
| AMB03.I1 | R | R | I |  | S83L |  |  |  |  |  |  |  |  |  |
| AMB03.I3 | R | I | I |  |  |  |  |  |  |  |  |  |  |  |
| AMB04.I2 | I | S | S | *qnrS1* |  |  |  |  |  |  |  |  |  |  |
| AMB31.I1 | R | R | R | *aac(6')-Ib-cr* | S83L | D87N |  |  |  | S80I |  |  |  |  |
| AMB32.I2 | R | I | I | *qnrS1* |  |  |  |  |  |  |  |  |  |  |
| AMB33.I2 | R | R | R |  | S83L | D87N |  |  |  | S80I |  |  |  |  |
| AMB06.I1 | R | I | I | *qnrS1* |  |  |  |  |  |  |  |  |  |  |
| AMB06.I2 | I | S | S | *qnrS1* |  |  |  |  |  |  |  |  |  |  |
| AMB34.I1 | R | S | I | *qnrS1* |  |  |  |  |  |  |  |  |  |  |
| AMB08.I3 | R | I | I | *qnrS1* |  |  |  |  |  |  |  |  |  |  |
| AMB09.I1 | I | S | S | *qnrS1* |  |  |  |  |  |  |  |  |  |  |
| AMB09.I2 | R | R | I | *qnrS1* |  |  |  |  |  |  |  |  |  |  |
| AMB10.I1 | R | I | I | *qnrS1* |  |  |  |  |  |  |  |  |  |  |
| AMB10.I2 | R | S | S |  | S83L |  |  |  | E62K |  |  |  |  |  |
| AMB11.I1 | R | I | I |  | S83L |  |  |  |  |  |  |  |  |  |
| AMB07.I2 | R | I | I | *qnrS1* |  |  |  |  |  |  |  |  |  |  |
| AMB35.I1 | R | I | I | *qnrS1* |  |  |  |  |  |  |  |  |  |  |
| AMB13.I1 | R | S | S | *qnrS1* |  |  | A56T |  |  |  |  |  |  |  |
| AMB13.I3 | R | R | R |  | S83L | D87N |  |  |  | S80I |  |  |  |  |
| AMB36.I3 | R | R | I |  | S83L |  |  |  |  | S80I |  |  |  |  |
| AMB14.I1 | R | R | I | *qnrS1* |  |  |  |  |  |  |  |  |  |  |
| AMB14.I2 | R | S | I | *qnrS1* |  |  |  |  |  |  |  |  |  |  |
| AMB37.I1 | I | S | S | *qnrS1* |  |  |  |  |  |  |  |  |  |  |
| AMB37.I2 | S | S | S | *qnrS1* |  |  |  |  |  |  |  |  |  |  |
| AMB15.I1 | R | I | I | *qnrS1* |  |  |  |  |  |  |  |  |  |  |
| AMB16.I1 | R | I | I | *qnrS1* |  |  |  |  |  |  |  |  |  |  |
| AMB16.I2 | R | S | S |  | S83L |  |  |  |  |  |  |  |  |  |
| AMB18.I1 | R | R | R | *aac(6')-Ib-cr (6')-Ib-cr* | S83L | D87N |  |  |  | S80I |  |  |  |  |
| AMB18.I3 | R | R | I | *qnrS1* |  |  |  |  |  |  |  |  |  |  |
| AMB19.I1 | R | R | R | *aac(6')-Ib-cr* | S83L | D87N |  |  |  | S80I |  |  |  |  |
| AMB19.I2 | R | I | S | *qnrS1* |  |  |  |  |  |  |  |  |  |  |
| AMB20.I1 | R | I | I |  | S83L |  | A56T |  |  |  |  |  |  |  |
| AMB20.I3 | R | I | S | *qnrS1* |  |  |  |  |  |  |  |  |  |  |
| AMB21.I1 | R | R | I | *qnrS1* |  |  |  |  |  |  |  |  |  |  |
| MAH29.C1 | R | R | I | *qnrS1* | S83A |  |  |  |  |  |  |  |  |  |
| MAH30.C1 | S | S | S |  |  |  |  |  |  |  |  |  |  |  |
| MAH31.C1 | R | R | R | *qnrS1* | S83L |  |  |  |  |  |  |  |  |  |
| MAH33.C1 | R | R | R |  | S83L | D87N |  |  |  | S80I | E84V |  |  |  |
| MAH69.C1 | I | I | S | *qnrS1* |  |  |  |  |  |  |  |  |  |  |
| MAH70.C1 | R | R | I | *qnrS1* |  |  |  |  |  |  |  |  |  |  |
| MAH71.C1 | I | I | I | *qnrS1* |  |  |  |  |  |  |  |  |  |  |
| MAH34.C1 | R | R | R |  | S83L | D87N |  |  |  |  |  |  |  | I529L |
| MAH73.C1 | R | S | S | *qnrS1* |  |  |  |  |  |  | E84V |  |  | I529L |
| MAH74.C1 | R | R | I | *qnrS1* | S83L |  |  |  |  |  |  |  |  |  |
| MAH75.C1 | R | R | R |  | S83L | D87N |  |  |  | S80I |  |  |  |  |
| MAH77.C1 | R | I | I | *qnrS1* |  |  |  |  |  |  |  |  |  |  |
| MAH78.C1 | R | R | R |  | S83L | D87N |  |  |  | S80I |  |  |  |  |
| MAH79.C1 | R | R | I | *qnrS1* |  |  |  |  |  |  |  |  |  |  |
| MAH82.C1 | R | R | R | *aac(6')-Ib-cr* | S83L | D87N |  |  |  | S80I |  |  |  |  |
| MAH50.C1 | R | I | S | *qnrS1* |  |  |  |  |  |  |  |  |  |  |
| MAH51.C1 | R | R | I | *qnrS1* |  |  | A56T |  |  |  |  |  |  |  |
| MAH52.C1 | R | I | S | *qnrS1* |  |  |  |  |  |  |  |  |  |  |
| MAH53.C1 | I | R | S | *qnrS1* |  |  |  |  |  |  |  |  |  |  |
| MAH84.C1 | S | S | S | *qnrS1* |  |  |  |  |  |  |  |  |  |  |
| MAH85.C1 | R | R | R | *qnrS1* | S83L |  |  |  |  | S80I |  |  |  |  |
| MAH86.C1 | R | I | I | *qnrS1* |  |  |  |  |  |  |  |  |  |  |
| MAH55.C1 | R | R | I | *qnrS1* |  |  |  |  |  |  |  |  |  |  |
| MAH90.C1 | I | I | S | *qnrS1* |  |  |  |  |  |  |  |  |  |  |
| MAH92.C1 | R | I | I | *qnrS1* |  |  |  |  |  |  |  |  |  |  |
| MAH03.C1 | R | R | R | *qnrS1* | S83L | D87N |  |  |  | S80I |  |  |  |  |
| MAH56.C1 | I | S | S | *qnrS1* |  |  |  |  |  |  |  |  |  |  |
| MAH93.C1 | R | S | S | *qnrS1* |  |  |  |  |  |  |  |  |  |  |
| MAH60.C1 | R | I | I | *qnrS1* |  |  |  |  |  |  |  |  |  |  |
| MAH100.C1 | R | I | I | *qnrS1* |  |  |  |  |  |  |  |  |  |  |
| MAH94.C1 | R | I | I | *qnrS1* |  |  |  |  |  |  |  |  |  |  |
| MAH96.C1 | R | I | I | *qnrS1* |  |  |  |  |  |  |  |  |  |  |
| MAH99.C1 | S | S | S | *qnrS1* |  |  |  |  |  |  |  |  |  |  |
| MAH18.C1 | I | S | S | *qnrS1* |  |  |  |  |  |  |  |  |  |  |
| MAH21.C1 | R | I | I | *qnrS1* |  |  |  |  |  |  |  |  |  |  |
| MAH23.C1 | R | R | I |  | S83L |  |  |  |  |  |  |  |  | I529L |
| MAH26.C1 | R | I | S | *qnrS1* |  |  |  |  |  |  |  |  |  |  |
| MAH61.C1 | R | R | R |  | S83L | D87N |  |  |  | S80I |  |  |  |  |
| MAH62.C1 | R | I | S | *qnrS1* |  |  |  |  |  |  |  |  |  |  |
| MAH64.C1 | R | I | I | *qnrS1* |  |  |  |  |  |  |  |  |  |  |
| MAH36.C1 | R | R | I | *qnrS1* |  |  |  |  |  |  |  |  |  |  |
| MAH37.C1 | R | R | R | *aac(6')-Ib-cr* | S83L | D87N |  |  |  | S80I |  |  |  |  |
| MAH39.C1 | R | R | I | *qnrS1* |  |  |  |  |  |  |  |  |  |  |
| MAH41.C1 | R | R | I | *qnrS1* |  |  |  |  |  |  |  |  |  |  |
| MAH43.C1 | R | I | I | *qnrS1* |  |  |  |  |  |  |  |  |  |  |
| MAH45.C1 | R | R | R |  | S83L | D87N |  |  |  | S80I |  |  |  |  |
| MAH47.C1 | R | R | R | *aac(6')-Ib-cr* | S83L | D87N |  |  |  | S80I |  |  |  |  |
| ANTb12.I1 | R | R | R | *qnrS1* | S83L |  |  |  |  |  |  |  |  |  |
| ANTb13.I1 | R | R | I | *qnrS1* | S83L |  |  |  |  |  |  |  |  |  |
| ANTb18.I1 | S | S | S | *qnrS1* |  |  |  |  |  |  |  |  |  |  |
| ANTb18.I2 | R | R | I | *qnrS1* | S83L |  |  |  |  |  |  |  |  |  |
| ANTb18.I3 | R | I | I | *qnrS1* |  |  |  |  |  |  |  |  |  |  |
| ANTb17.I1 | R | I | I | *qnrS1* |  |  |  |  |  |  |  |  |  |  |
| ANTb21.I1 | R | I | I | *qnrS1* |  |  |  |  |  |  |  |  |  |  |
| ANTb22.I1 | R | I | I | *qnrS1* |  |  |  |  |  |  |  |  |  |  |
| ANTb23.I1 | R | I | S | *qnrS1* |  |  |  |  |  |  |  |  |  |  |
| ANTb23.I2 | R | R | R |  | S83L | D87N |  |  |  | S80I | E84V |  |  |  |
| ANTb27.I1 | R | R | R |  | S83L | D87N |  |  |  | S80I |  |  |  |  |
| ANTb29.I1 | R | I | I | *qnrS1* |  |  |  |  |  |  |  |  |  |  |
| ANTb33.I1 | R | I | S | *qnrS1* |  |  |  |  |  |  |  |  |  |  |
| ANTb35.I1 | R | R | R | *aac(6')-Ib-cr* | S83L | D87N |  | S57T |  | S80I |  |  |  |  |
| ANTb36.I1 | S | S | S |  |  |  |  |  |  |  | E84V |  |  | I529L |
| ANTb52.I1 | I | S | S | *qnrS1* |  |  |  |  |  |  |  |  |  |  |
| ANTb52.I2 | R | I | S | *qnrS1* |  |  |  |  |  |  |  |  |  |  |
| ANTb58.I1 | R | S | S | *qnrS1* |  |  |  |  |  |  |  |  |  |  |
| ANTb02.I1 | I | S | S | *qnrS1* |  |  |  |  |  | S80I |  |  |  |  |
| ANTb02.I2 | R | I | I | *qnrS1* |  |  |  |  |  |  |  |  |  |  |
| ANTb06.I1 | R | R | R |  | S83L | D87N |  |  |  | S80I |  |  |  |  |
| ANTb07.I1 | S | S | S | *qnrS1* |  |  |  |  |  |  |  |  |  |  |
| ANTb08.I1 | I | S | S |  | S83L |  |  |  |  |  |  |  |  |  |
| ANTb09.I1 | R | I | I |  |  |  |  |  |  |  |  |  |  |  |
| ANTa03.I1 | R | R | R |  | S83L | D87N |  |  |  | S80I |  |  |  |  |
| ANTa06.I1 | R | I | I | *qnrS1* |  |  |  |  |  |  |  |  |  |  |
| ANTa11.I1 | R | R | R | *aac(6')-Ib-cr* | S83L | D87N |  |  |  | S80I |  |  | S458T |  |
| ANTa12.I1 | R | R | R | *aac(6')-Ib-cr* | S83L | D87N |  |  |  |  | E84V | A192V |  |  |
| ANTa14.I1 | R | R | I | *qnrS1* |  |  |  |  |  |  |  |  |  |  |
| ANTa14.I3 | R | R | R | *aac(6')-Ib-cr* | S83L | D87N |  |  |  | S80I |  |  |  |  |
| ANTa07.I1 | R | R | R |  | S83L | D87N |  |  |  | S80I |  |  |  |  |
| ANTa08.I2 | I | I | S | *qnrS1* |  |  |  |  |  |  |  |  | S458T |  |
| ANTa21.I3 | R | R | R | *aac(6')-Ib-cr* | S83L | D87N |  |  |  | S80I |  | A192V |  | I529L |
| ANTa22.I2 | R | R | R |  | S83L | D87N |  |  |  | S80I |  |  |  |  |
| ANTa23.I1 | R | I | I | *qnrS1* |  |  |  |  |  |  |  |  |  |  |
| ANTa25.I2 | S | S | S |  |  |  |  |  |  |  |  |  |  |  |
| ANTa32.I2 | R | I | S |  |  |  |  |  |  |  |  |  |  |  |
| ANTa32.I3 | R | R | I | *qnrS1* |  |  |  |  |  |  |  |  |  |  |
| ANTa36.I1 | R | R | R | *aac(6')-Ib-cr* | S83L | D87N |  |  |  | S80I |  |  | S458T |  |
| ANTa36.I2 | R | R | R |  | S83L | D87N |  |  |  | S80I |  |  |  |  |
| ANTa39.I2 | R | R | I | *qnrS1* |  |  |  |  |  |  |  |  |  |  |
| ANTa43.I1 | R | R | R |  | S83L | D87N |  |  |  | S80I |  |  |  |  |
| ANTa43.I4 | I | I | S | *qnrS1* |  |  |  |  |  |  |  |  |  |  |
| ANTa47.I1 | R | R | R |  | S83L | D87N |  |  |  | S80I |  |  | S458T |  |
| ANTa47.I2 | S | R | R |  |  |  |  |  |  |  |  |  |  |  |
| ANTa50.I3 | R | R | R |  | S83L | D87N |  |  |  | S80I |  |  |  |  |
| ANTa51.I3 | R | I | I | *qnrS1* |  |  |  |  |  |  |  |  |  |  |
| ANTa60.I2 | R | I | S | *qnrS1* |  |  |  |  |  |  |  |  |  |  |
| ANTa67.I1 | R | R | I | *qnrS1* |  |  |  |  |  |  |  |  |  |  |
| ANTa70.I1 | R | R | I | *qnrS1* | S83A |  |  |  |  |  |  |  |  |  |
| ANTa71.I2 | I | S | S | *qnrS1* |  |  |  |  |  |  |  |  |  |  |
| ANTa72.I1 | R | R | R | *qnrS1* |  |  |  |  |  |  |  |  |  |  |
| ANTa72.I2 | R | R | R | *aac(6')-Ib-cr* | S83L | D87N |  |  |  | S80I |  |  | S458T |  |
| ANTa72.I3 | S | S | S |  |  |  |  |  |  |  |  |  |  |  |
| ANTa74.I1 | R | R | R |  | S83L | D87N |  |  |  | S80I |  |  |  |  |
| ANTa82.I1 | I | S | S | *qnrS1* |  |  |  |  |  |  |  |  |  |  |
| ANTa84.I1 | R | R | R | *aac(6')-Ib-cr* | S83L | D87N |  |  |  | S80I |  |  |  |  |
| ANTa96.I1 | S | S | S |  |  |  |  |  |  |  |  |  | S458T |  |
| ANTa98.I1 | I | S | S | *qnrS1* |  |  |  |  |  |  |  |  |  |  |
| ANTa01.I1 | R | R | R | *aac(6')-Ib-cr* | S83L | D87N |  |  |  | S80I |  |  |  |  |
| TOA10.C1 | R | R | I | *qnrS1* |  |  |  |  |  |  |  |  |  |  |
| TOA30.C1 | R | R | R | *aac(6')-Ib-cr* | S83L | D87N |  |  |  | S80I |  |  |  |  |
| TOA43.C1 | R | R | I | *qnrS1* |  |  |  |  |  |  |  |  |  |  |
| TOA28.C1 | R | R | R |  | S83L | D87N |  |  |  | S80I |  |  |  |  |
| TOA53.C1 | R | R | I | *qnrS1* |  |  |  |  |  |  |  |  |  |  |
| ANTc61.I1 | R | R | I | *qnrS1* |  |  |  |  |  |  |  |  |  |  |
| ANTc20.I1 | R | R | I | *qnrS1* |  |  |  |  |  |  |  |  |  |  |
| ANTc68.I1 | R | R | I | *qnrS1* |  |  |  |  |  |  |  |  |  |  |
| ANTc68.I2 | R | R | S | *qnrS1* |  |  |  |  |  |  |  |  |  |  |
| ANTc09.I1 | R | R | I | *qnrS1* |  |  |  |  |  |  |  |  |  |  |
| ANTc33.I1 | R | I | I | *qnrS1* |  |  |  |  |  |  |  |  |  |  |
| ANTc37.I1 | R | R | I |  | S83L |  | A56T |  |  |  |  |  |  |  |
| ANTc40.I1 | R | R | R |  | S83L | D87N |  |  |  | S80I |  |  |  |  |
| ANTc44.I1 | R | R | R | *aac(6')-Ib-cr* | S83L | D87N |  |  |  | S80I |  |  |  |  |
| ANTc49.I1 | R | R | I | *qnrS1* |  |  |  |  |  |  |  |  |  |  |
| ANTc56.I3 | R | R | I | *qnrS1* |  |  |  |  |  |  |  |  |  |  |
| ANTc59.I2 | R | R | R | *qnrS1* | S83L | D87N |  |  |  | S80I |  |  |  |  |
| ANTc66.I3 | R | R | I | *qnrS1* |  |  |  |  |  |  |  |  |  |  |
| ANTc70.I1 | R | R | I | *qnrS1* |  |  |  |  |  |  |  |  |  |  |
| ANTc72.I1 | R | R | R | *aac(6')-Ib-cr* | S83L | D87N |  |  |  | S80I |  |  |  |  |
| ANTc83.I1 | R | I | I | *qnrS1* |  |  |  |  |  |  |  |  |  |  |
| ANTc91.I2 | R | I | I | *qnrS1* |  |  |  |  |  |  |  |  |  |  |
| ANTc95.I1 | R | S | S |  | S83L |  |  |  |  |  |  |  |  |  |
| ANTc96.I1 | R | I | S | *qnrS1* |  |  |  |  |  |  |  |  |  |  |
| ANTc99.I1 | R | R | S | *qnrS1* |  |  |  |  |  |  |  |  |  |  |
| ANTc19.I1 | R | R | I | *qnrS1* |  |  | A56T |  |  |  |  |  |  |  |
| ANTc19.I2 | R | R | I | *qnrS1* |  |  |  |  |  |  |  |  |  |  |
