# Supplementary material for: Prevalence, Risk Factors, and Genetic Characterization of Extended-Spectrum Beta-Lactamase Escherichia coli Isolated From Healthy Pregnant Women in Madagascar
Source: Front Microbiol. 2021 Dec 24;12:786146. doi: 10.3389/fmicb.2021.786146 (PMC8740230; doi:10.3389/fmicb.2021.786146)
Supplement: Supplementary file 7 [file Table_3.DOCX]

**Table S3.** Frequency of clonal complex (STc) determined according to the Achtman scheme.

**No STc:** STc not defined in Enterobase (<http://enterobase.warwick.ac.uk/species/index/ecoli>) for the corresponding ST. **Unknown** – ST and STc not defined in Enterobase.

| **STc** | **N (%)** | **Phylogroup** |
| --- | --- | --- |
| 10 | 59 (35.1) | A |
| 155 | 11 (6.6) | B1 |
| 38 | 7 (4.2) | D |
| 446 | 6 (3.6) | B1 |
| 46 | 5 (3.0) | A |
| 398 | 5 (3.0) | A |
| 131 | 5 (3.0) | B2 |
| 206 | 3 (1.8) | A |
| 226 | 3 (1.8) | A |
| 23 | 3 (1.8) | C |
| 394 | 3 (1.8) | D |
| 168 | 2 (1.2) | A |
| 32 | 1 (0.6) | A |
| 156 | 1 (0.6) | B1 |
| 469 | 1 (0.6) | B1 |
| 522 | 1 (0.6) | A |
| 648 | 1 (0.6) | F |
| No STc | 41 (24.4) | - |
| Unknown | 10 (6.0) | - |
