# Supplementary material for: Prevalence, Risk Factors, and Genetic Characterization of Extended-Spectrum Beta-Lactamase Escherichia coli Isolated From Healthy Pregnant Women in Madagascar
Source: Front Microbiol. 2021 Dec 24;12:786146. doi: 10.3389/fmicb.2021.786146 (PMC8740230; doi:10.3389/fmicb.2021.786146)

Figure S1. Geographical repartition of ESBL-*E. coli* isolates in the capital. Geographical distribution of ESBL-*E. coli* sequence types identified in participants from the capital Antananarivo. Locations are limited to the city quarter level to preserve anonymity of participants.

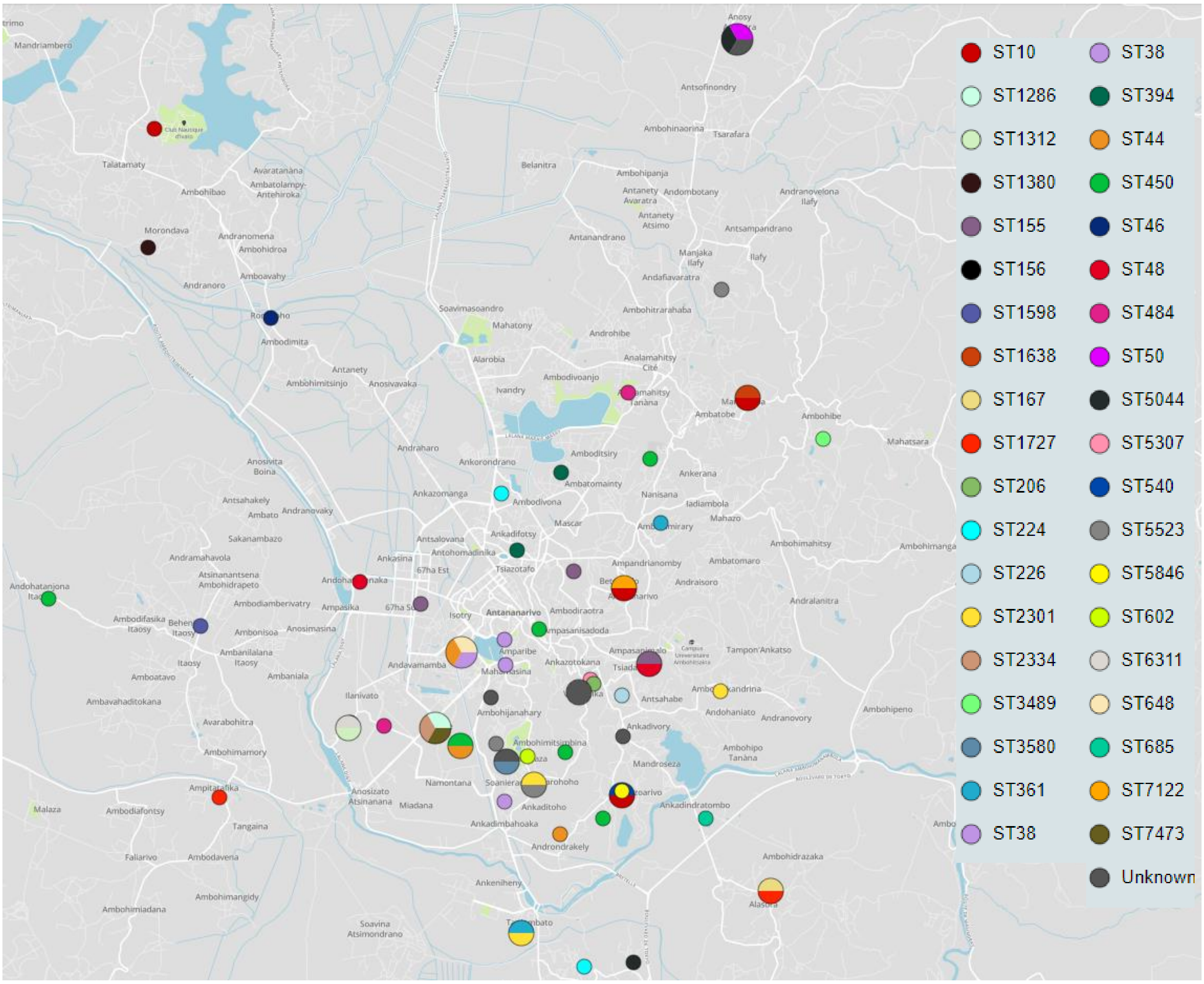

Supplement: Supplementary file 1 [file Data_Sheet_1.PDF]
